# Ensemble transformer with post-hoc explanations for depression emotion and severity detection

**DOI:** 10.1016/j.isci.2025.114605

**Published:** 2026-01-05

**Authors:** Sazzadul Islam, Rezaul Haque, Mahbub Alam Khan, Arafath Bin Mohiuddin, Md Ismail Hossain Siddiqui, Zishad Hossain Limon, Katura Gania Khushbu, S M Masfequier Rahman Swapno, Md. Redwan Ahmed, Abhishek Appaji

**Affiliations:** 1 Department of Computer Science and Engineering, BRAC University, Dhaka 1212, Bangladesh; 2 Department of Computer Science and Engineering, East West University, Dhaka 1212, Bangladesh; 3 Department of Management Information System, Pacific State University, 3424 Wilshire Boulevard, 12th Floor, Los Angeles, CA 90010, USA; 4 Department of Engineering and Technology, Westcliff University, Irvine, CA 92614, USA; 5 Engineering/Industrial Management, Westcliff University, Irvine, CA 92614, USA; 6 Department of Computer Science, Westcliff University, Irvine, CA 92614, USA; 7 Department of Computer Science and Engineering, Bangladesh University of Business and Technology, Dhaka 1216, Bangladesh; 8 Department of Medical Electronics Engineering, B.M.S. College of Engineering, Bull Temple Road, Bengaluru, Karnataka 560019, India; 9 Maastricht University, University Eye Clinic Maastricht, Minderbroedersberg 4-6, Maastricht, the Netherlands

**Keywords:** Artificial intelligence, Psychology

## Abstract

This study presents an ensemble transformer framework for detecting depression-related emotions and classifying their severity in social media text. It addresses the need for scalable and trustworthy AI solutions in mental health by integrating four transformer models. The DepTformer-XAI-SV model uses a weighted soft-voting mechanism based on validation macro-F1 scores to improve accuracy and incorporates LIME to highlight key linguistic features associated with depression. The framework is evaluated on two benchmark datasets: DepressionEmo, with eight emotion classes, and the merged depression severity detection (MDSD), with four severity levels, both sourced from social media. To address class imbalance, we use class-weighted cross-entropy, stratified k-fold splits, and minority-aware sampling. Results show that the model surpasses individual transformer models and traditional methods, achieving macro-F1 scores of 80.44% for DepressionEmo and 79.88% for MDSD, significantly improving minority class detection. Lastly, a web application has been developed for interactive and interpretable inference.

## Highlights

• Ensemble transformers boost depression emotion and severity detection accuracy
• Class imbalance handled with weighted losses, stratified splits, and sampling
• LIME-based token explanations validated with deletion and stability metrics
• Web app enables real-time, interpretable predictions for mental-health text

## Graphical abstract

**Figure:**
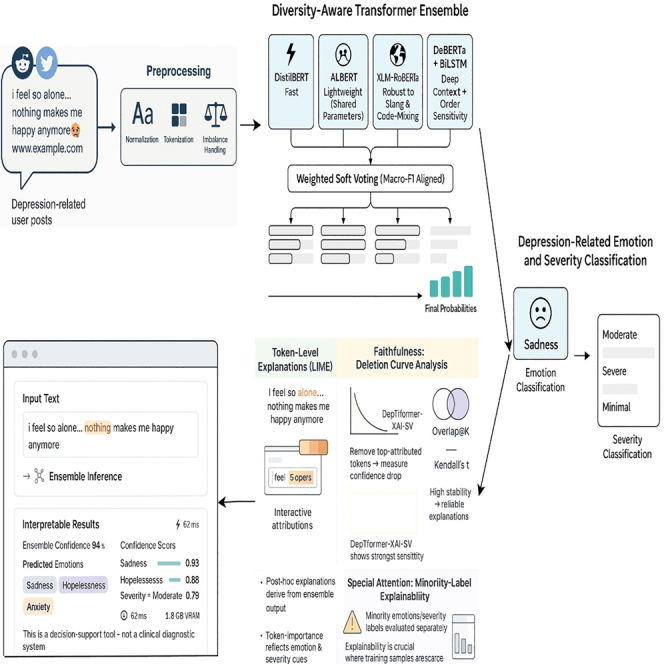

## Introduction

Depression imposes considerable personal and economic burdens worldwide. The World Economic Forum^1^ estimates that productivity losses due to depression reach trillions of USD annually, highlighting the need for scalable, early detection and support. Clinically, depressive episodes are characterized by persistent low mood, anhedonia, and cognitive or psychomotor changes that can impair functioning. For instance, individuals with major depressive disorder exhibit significantly reduced workplace productivity compared to those without the disorder.^2^

The diagnostic presentation of depression varies across cultural and resource contexts,^3^^,^^4^^,^^5^ complicating case identification and the assessment of symptom severity. Although the DSM-5 and ICD-11 criteria aim to reduce over- and under-diagnosis by focusing on impairment and severity,^6^^,^^7^ adherence in practice is inconsistent. Previous studies^8^ suggest that using structured criteria and severity anchors can greatly improve diagnostic accuracy and minimize misclassifications. Moreover, social and economic stressors, such as unemployment, are correlated with a higher burden of depressive symptoms.^9^^,^^10^ The severity of depression can vary widely, with a subset of cases carrying an increased risk of suicide.^11^^,^^12^ Social media platforms provide large datasets in which individuals sometimes express their distress,^13^^,^^14^^,^^15^ creating opportunities for population-scale monitoring and research. Posts can include both explicit and indirect expressions of distress (e.g., metaphors or withdrawal cues), resulting in linguistically rich yet noisy signals.^16^^,^^17^^,^^18^ Any computational analysis of this data must prioritize de-identification, comply with platform terms, and refrain from making diagnostic claims.

Recent research on automated depression analysis includes emotion detection, severity classification, transformer-based ensembles,^19^^,^^20^^,^^21^^,^^22^ and explainability. While these systems often achieve strong benchmark performance, they generally struggle with generalizability, interpretability, and real-world deployment readiness. Early emotion detection methods used CNN-RNN hybrids. For instance, Machová et al.^23^ achieved 91% accuracy with Conv1D-LSTM,^15^ and Thekkekara et al.^24^ applied CNN-BiLSTM with attention on eRisk, but neglected data imbalance and contextual cues. Transformer-based models showed higher accuracy: Kodati et al.^25^ reached 91.2% with XLNet during COVID-19 but lacked cross-context robustness. Jamali et al.^26^ had an F1 score of 97.44% on binary Twitter data but faltered in multi-label scenarios. In another study, Rahman et al.^27^ introduced DepressionEmo with BART, achieving an F1-Macro of 0.76, yet imbalances persisted. Performance improvements were noted by Violides et al.^28^ with RoBERTa, while Khan et al.’s^29^ contrastive learning approach required high computational resources and struggled on smaller datasets. Most emotion-focused studies remain binary or multi-class, often overlooking interpretability and deployment needs.

In severity classification, Kabir et al.^30^ employed BiGRU on Bengali posts with an accuracy of 81%, but faced challenges with code-mixing and data scale. Further, Muñoz et al.^31^ combined lexicon and transformer embeddings, scoring F1 74.38% and 63.94%, respectively, but had issues with label skew. Similarly, Burdisso et al.^32^ used SS3 to predict BDI scores, although sensitivity to small sample sizes undermined robustness. DEPTWEET^33^ reached an AUC of 88.6% using DistilBERT, but faced annotation subjectivity and cultural biases. In another study, Arachchige et al.^34^ achieved 79% F1 with LSTM on a small sample of 2,140 without addressing imbalance. Key issues include dataset diversity, limited linguistic coverage, and a lack of explainability. Ensemble methods have been explored to improve robustness. Thiab et al.^35^ combined CNN, BERT, XLNet, and RoBERTa (F1 77.07%), yet their voting scheme lacked adaptive weighting. Similarly, Tavchioski et al.^36^ evaluated GMT ensembles with varying F1 scores but struggled under domain shifts. Singh et al.^37^ achieved 95.01% accuracy using attention-enabled ensembles on 2.3M samples, facing high computational demands. Further, Ogunleye et al.^38^ incorporated sentiment into Sentence-BERT ensembles (76% F1), and Nijhawan et al.^39^ combined BERT with LDA and RF (83% F1) but lacked scalability. GPT-4 chatbot analysis by Arriba-Pérez and García-Méndez^40^ reached 91% F1 but was limited to small dialogue datasets. Few ensemble systems utilize principled fusion strategies or provide explanations suited for clinical applications.

Recent research has increasingly incorporated XAI techniques to improve transparency in mental-health NLP systems. Chowdhury et al.^41^ applied SHAP to BanglaBERT and DepGPT, achieving strong performance (F1: 0.9804) but at high computational cost. Bao et al.^42^ evaluated WT5 and T5+BERT on PsySym and BDI-Sen, reaching 95% F1 with expert validation, though results varied across datasets. Ahmed et al.^43^ combined LIME with BERT-based ensembles on DEPTWEET, improving AUC-ROC by 13.5%, yet platform constraints limited generalizability. Imans et al.^44^ proposed a layered SHAP-based ensemble attaining 88.33% accuracy, but architectural complexity hindered real-time deployment. XAI has also been applied to broader mental-health detection: Kerz et al.^45^ used BiLSTM and MentalRoBERTa across SMHD, Dreaddit, GoEmotions, and MBTI, achieving F1 scores of 57–70% and 81.6%, but relied heavily on Reddit data and binary labels. Masud et al.^46^ predicted depression among Bangladeshi students using demographic and academic features, obtaining 91.1% accuracy with Random Forest and 98.6% recall with RoBERTa, yet faced demographic bias and small-sample limitations. Similarly, Imans et al.^44^ combined BiLSTM, attention, and SHAP for depression detection on DAIC-WOZ and Twitter, reporting 92.4% accuracy but limited multilingual and cross-platform robustness. In suicide-risk assessment, Tang et al.^47^ used SHAP with classical models on augmented medical records, achieving 97% accuracy but drawing from a small, tabular dataset. Zulfiker et al.^48^ built a DL + XAI framework using 20k Reddit posts, attaining 93.39% accuracy, though sarcasm, cultural bias, and English-only data remained challenges. Oliveira et al.^49^ studied MHP trust in XAI-enabled systems and found that LIME explanations and misclassifications reduced trust.

Across prior studies, major gaps persist: (i) limited generalization due to single-task or single-dataset focus; (ii) insufficient handling of class imbalance and inconsistent reporting of macro-averaged metrics; (iii) ensembles without principled, validation-driven weighting; and (iv) weak integration of explainability into both modeling and deployment. Our work addresses these issues by combining multi-label emotion detection with multi-class severity classification using diverse datasets, employing a diversity-aware transformer ensemble with validation-weighted fusion, actively mitigating imbalance, and providing validated token-level explainability with efficient deployment. Transformer encoders are effective for capturing long-range and contextual dependencies in noisy text.^50^^,^^51^ At the same time, ensembling techniques can reduce variance and improve robustness across different linguistic styles.^52^ However, real-world applicability faces ongoing challenges, including class imbalance, linguistic ambiguity (including idioms, sarcasm, and hyperbole), and computational constraints that limit throughput and memory when processing long sequences. For example, large language models have high accuracy but at the price of high computation. Furthermore, these models often struggle with handling long text sequences due to input token length limitations, which can result in truncated context and diminished overall performance. Additionally, there is a growing expectation for explainability in clinical-adjacent environments,^53^^,^^54^ yet post-hoc tools are inconsistently validated, and ensemble methods may obscure decision pathways. Furthermore, the transition from AI research to real-world applications remains limited.^55^ Although some research^7^^,^^40^ efforts have proposed visualization tools, few systems provide reports on latency, energy consumption, alongside accuracy, or present interpretable user interfaces.

This study aims to develop a data-driven system for analyzing depression that addresses existing limitations by integrating imbalance-sensitive training, a diversity-aware transformer ensemble, and validated explainability with deployment-focused reporting. The specific objectives are as follows.

• To achieve competitive, validated performance in imbalanced multi-label emotion detection and multi-class severity prediction.
• Show statistically significant macro-F1 improvements of the proposed ensemble over the single backbone architectures on both tasks/datasets (paired tests over k folds, 95% CI), with a pre-specified target of ≥1.0 percentage points (pp) overall and ≥2.0 pp on minority labels.
• Validate token-level XAI beyond visuals by (i) the deletion of AUC trends for K∈5,10, (ii) stability across resamplings with Overlap@ K≥0.60 and Kendall’s τ≥0.50 (minority labels reported explicitly), and (iii) ablations of perturbation policies (mask vs. delete).
• Develop an interpretable web prototype and report on throughput to inform real-world trade-offs.

**Figure 1:**
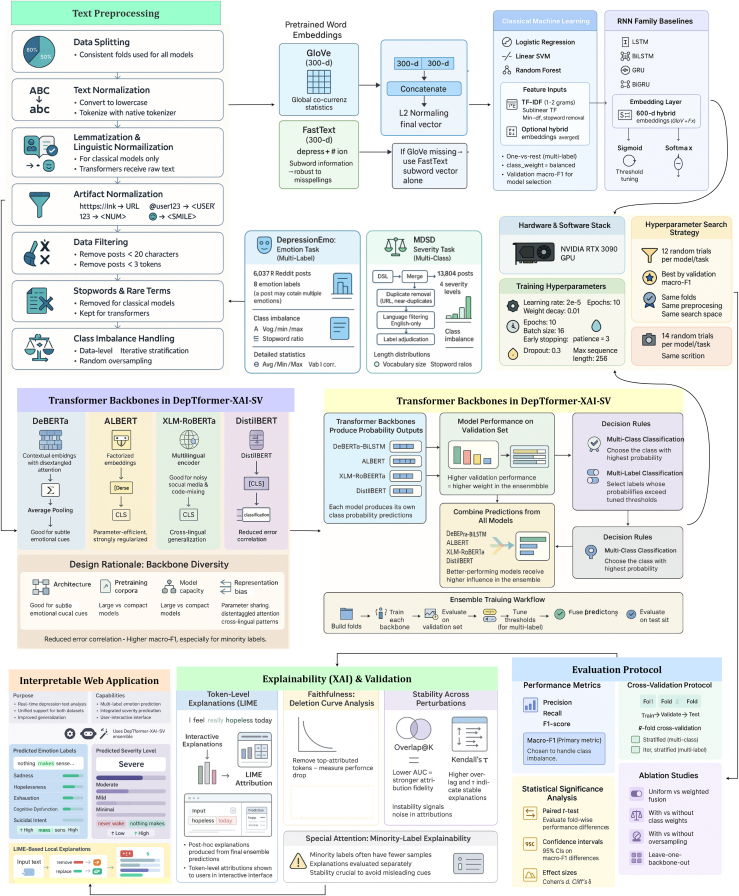
Overview of the proposed methodology for depression detection using social media text

**Figure 2:**
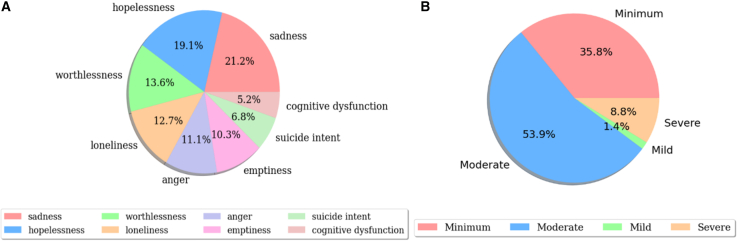
Class distribution of DepressionEmo and MDSD dataset

**Figure 3:**
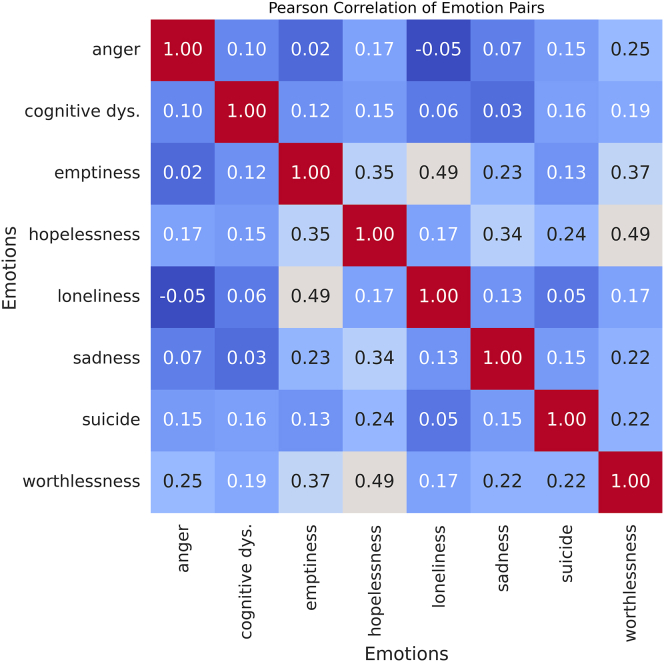
Pearson correlation between emotion labels in the multi-label DepressionEmo dataset Warmer colors indicate higher co-occurrence across posts (e.g., worthlessness-hopelessness and loneliness-emptiness co-occur frequently), while Anger shows weaker correlations with inward-facing emotions.

**Figure 4:**
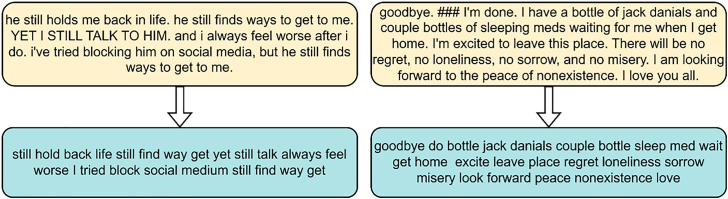
Sample text output when an input text goes through our preprocessing pipeline

**Figure 5:**
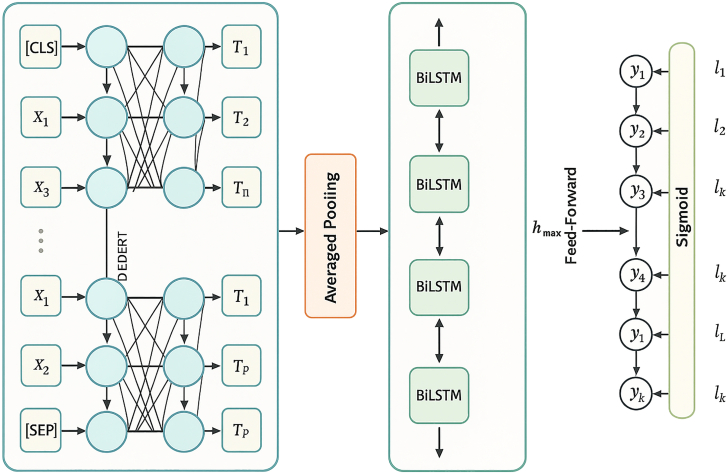
DeBERTa processes the input text sequence first Then the contextual embeddings are averaged with an average pooling layer to produce a set of pooled features. Then, we input the pooled features into a BiLSTM layer. A feedforward layer with sigmoid activation is used for the classification, passing through the final hidden states.

**Figure 6:**
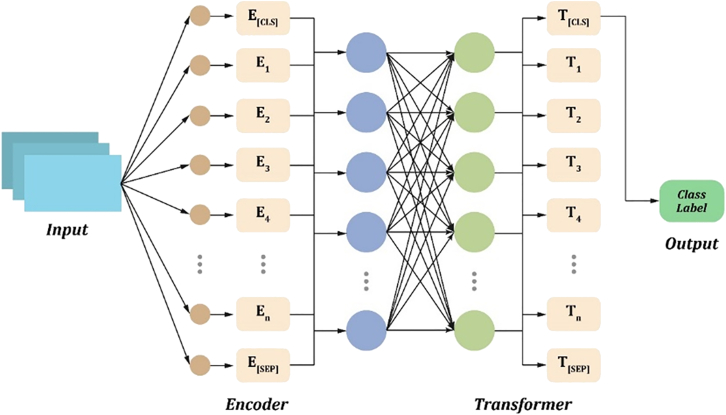
ALBERT architecture for text classification It tokenizes the input sequence and generates token embeddings through an encoder. These embeddings are processed by transformer layers to understand contextual relationships and semantic dependencies.

**Figure 7:**
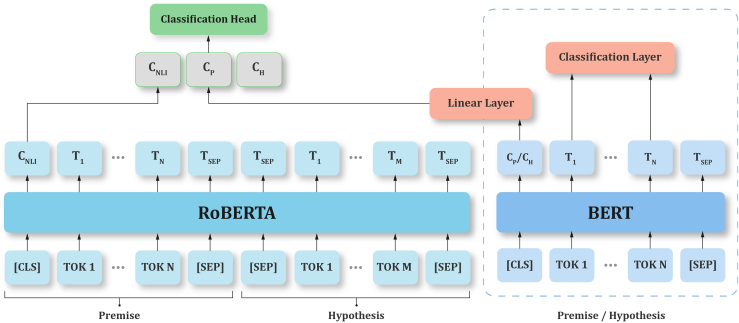
Xlm-ReBERTa architecture for multilingual text classification It processes paired input sequences, such as premise and hypothesis, using RoBERTa for feature extraction.

**Figure 8:**
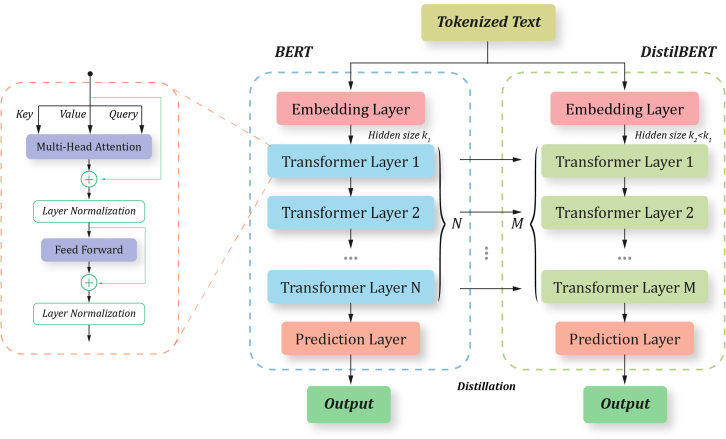
DistilBERT architecture for efficient text classification Key components such as multi-head attention, layer normalization, and feedforward mechanisms demonstrate the transformer’s functionality. It reduces the number of transformer layers (M < N) while retaining essential features through knowledge distillation. The embedding layer converts tokenized text into contextual embeddings, which are then processed through fewer transformer layers for enhanced efficiency.

**Figure 9:**
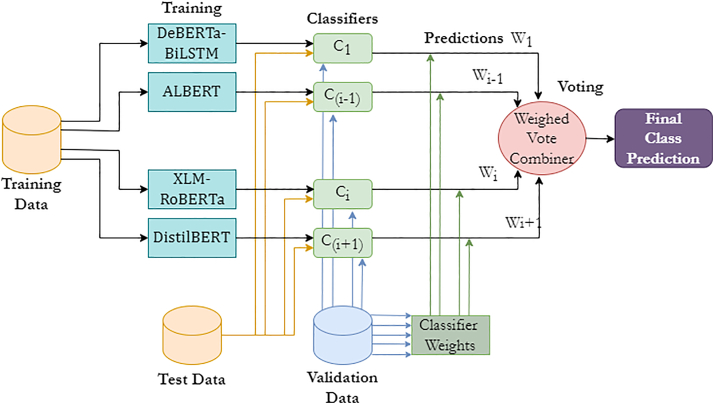
DepTformer-XAI-SV model for depression analysis The training data are processed through multiple transformer-based models to generate predictions. Each classifier is evaluated on validation data to determine weights based on its performance. During inference, predictions from these classifiers are combined using a weighted majority voting strategy, allowing higher-performing models to influence the final class prediction more.

To address these objectives, we employed two datasets: the DepressionEmo dataset, which contains 6,037 Reddit posts labeled across eight emotion categories, and the Depression Severity Levels dataset, which consists of 13,804 posts categorized into four severity levels. Our preprocessing steps included text normalization, tokenization, and the generation of adequate word embedding techniques. We train four heterogeneous transformers—DistilBERT, ALBERT, XLM-RoBERTa, and DeBERTa with a BiLSTM head—and fuse their probabilities via validation-weighted soft voting aligned to macro-F1. For interpretability, we used LIME to get token-level attributions (post-hoc to the fused model), and we validated them with token-deletion curves plus stability metrics. Additionally, we created a real-time web application that combines the ensemble model with the outputs from LIME. This application enables users to input text, receive predictions for both emotion and severity, and visualize token importance. An overview of the pipeline and model components is shown in Figure 1, dataset details in Figures 2, 3, and 4 baseline model architectures in Figures 5, 6, 7, and 8 and the proposed model architecture in Figure 9. Our contributions are as follows.

• A diversity-aware transformer ensemble with validation-weighted fusion that improves macro and micro- F1, especially on minority labels.
• An imbalance-sensitive training recipe (class weights, iterative stratification, capped oversampling, per-class thresholds) with ablation evidence.
• A faithfulness/stability evaluation protocol for token-level explanations (${\text{AUC}}_{\text{del}}$, Overlap@K, Kendall’s τ) that goes beyond visuals.
• A deployment-minded report of latency/VRAM/energy, along with an interpretable web prototype to support practice translation by presenting an accuracy-cost Pareto analysis.

## Result

### Text analysis

**Figure 10:**
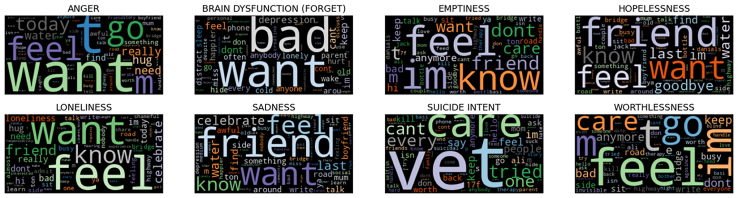
Wordcloud representation of the DepressionEmo dataset after performing preprocessing steps

**Figure 11:**
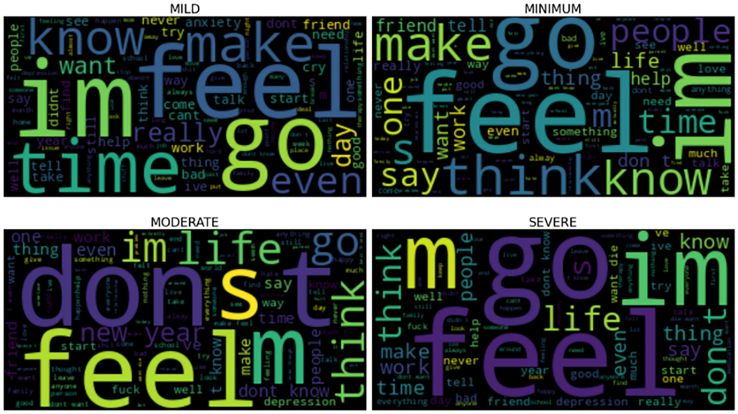
Wordcloud of DSL dataset after performing preprocessing steps

To analyze the linguistic characteristics of both datasets, we generated word clouds for each emotion and severity category. Figure 10 displays examples from the DepressionEmo dataset. In the anger class, we see words such as “go,” “want,” and “bad,” which reflect impulsive reactions and feelings of frustration. The cognitive dysfunction category includes frequent words such as “bad,” “want,” and “keep,” highlighting a self-critical inner dialogue and difficulties in maintaining focus. The emptiness category features terms such as “feel,” “don’t,” and “know,” which convey feelings of detachment and uncertainty. Similarly, the hopelessness word cloud contains “friend,” “last,” and “goodbye,” representing despair and social withdrawal. In the sadness category, words such as “friend,” “feel,” and “know” emphasize the importance of relationships in emotional well-being. Lastly, the worthlessness and suicidal intent categories are characterized by words such as “care,” “feel,” and “can’t,” which express deep emotional distress and a loss of self-worth. For the DSL dataset (Figure 11), the word clouds reveal distinct lexical trends across severity levels. In the mild category, common terms such as “feel,” “go,” “time,” and “day” suggest engagement with daily life and reflective thinking. The Moderate category introduces words such as “don’t,” “feel,” “life,” and “think,” indicating an increase in existential concerns and emotional tension. In the severe category, the words “feel,” “don’t,” and “life” dominate, emphasizing emotional withdrawal and distress. Overall, as severity increases, there is a linguistic shift from action-oriented terms (such as “time” and “go”) to more introspective and negative expressions (such as “don’t” and “life”), mirroring the escalating impact of depressive symptoms.

### Performance comparison across evaluation metrics

**Figure 12:**
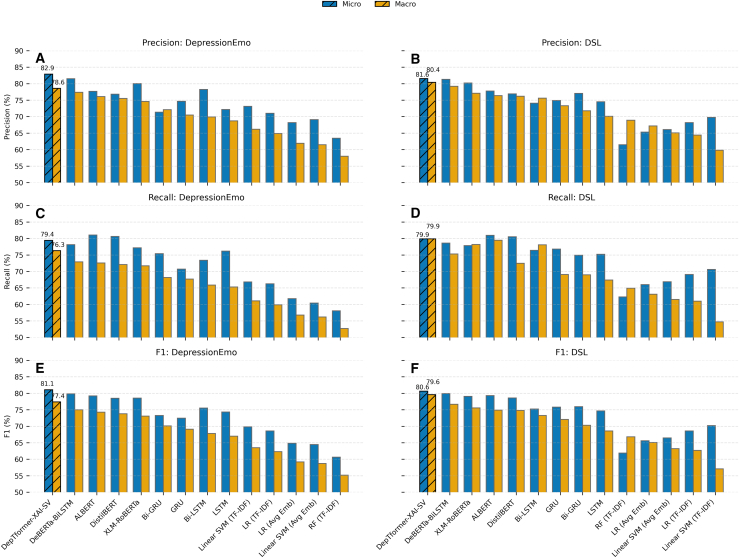
Micro- and macro-averaged precision, recall, and F1-score comparison across all models on DepressionEmo and DSL (A, C, and E) show Precision, Recall, and F1 scores for DepressionEmo, while panels (B, D, and F) display the same metrics for DSL. The bars represent mean test performance (blue for micro, orange for macro), and the models are ranked by macro-F1 for each dataset. The proposed DepTformer-XAI-SV is marked with hatched bars.

Figure 12 shows the DepTformer-XAI-SV model consistently outperforms other models across both datasets, effectively balancing performance metrics. Transformer-based models also demonstrate strong performance, exceeding that of traditional deep learning models. The DSL dataset shows slightly higher precision and F1 scores, suggesting it contains more distinguishable patterns compared to the DepressionEmo dataset.

The results in Table 1 show significant differences in classifier performance for multi-label classification tasks. The DepTformer-XAI-SV model outperforms all transformer baselines, achieving the highest scores in both micro- and macro-averaged metrics and providing balanced generalization across frequent and rare emotion labels. DeBERTa-BiLSTM is the second-best performer (micro-F1 = 79.85, macro-F1 = 75.0), surpassing single-encoder models such as ALBERT and XLM-RoBERTa, demonstrating the advantages of combining contextualized embeddings with recurrent post-processing. ALBERT has a strong recall of 81.04, indicating sensitivity to emotional cues, but its moderate precision of 77.67 shows overactivation on ambiguous expressions. XLM-RoBERTa has slightly better precision but lower recall, suggesting it may miss subtle emotional signals. DistilBERT, while efficient, lags behind larger models, indicating that parameter compression can impair performance in multi-label emotion detection. RNN models such as Bi-LSTM and Bi-GRU show moderate results. They capture sequential dependencies but do not achieve the contextual depth of pre-trained transformers. Their precision-recall trade-offs reflect a tendency to overfit on dominant emotion categories, especially in imbalanced datasets without task-specific pretraining. Unidirectional GRU/LSTM models perform even worse. Classical machine learning models score a lower macro-F1 score of less than 65. TF-IDF features lack semantic depth, and averaging embeddings does not effectively convey emotional nuances. The SVM with TF-IDF achieves a micro-F1 of 69.8, confirming that while lexical signals provide some discrimination, they fall short of capturing the complex sentiment dynamics required for multi-label tasks.

**Table 1:** Performance comparison of multi-label classifiers on the DepressionEmo test set using micro- and macro-averaged metrics

Model	Name	Micro-Averaged Metrics			Macro-Averaged Metrics		
		Precision	Recall	F1 Score	Precision	Recall	F1 Score
Proposed	DepTformer-XAI-SV	82.91	79.42	81.08	78.6	76.3	77.4
Transformers	DistilBERT	76.84	80.63	78.52	75.5	72.1	73.8
	XLM-RoBERTa	80.03	77.20	78.55	74.6	71.7	73.1
	ALBERT	77.67	81.04	79.21	76.1	72.6	74.3
	DeBERTa-BiLSTM	81.49	78.12	79.85	77.4	72.9	75.0
DL	Bi-GRU	71.37	75.41	73.29	72.1	68.2	70.1
	Bi-LSTM	78.23	73.41	75.52	69.9	65.9	67.8
	GRU	74.66	70.71	72.48	70.5	67.7	69.1
	LSTM	72.18	76.19	74.33	68.7	65.3	67.0
ML	Linear SVM (OvR, TF–IDF)	73.12	66.85	69.84	66.2	61.1	63.5
	LR (OvR, TF–IDF)	71.04	66.27	68.57	64.9	59.9	62.3
	RF (TF–IDF)	63.45	58.09	60.65	58.0	52.7	55.2
	Linear SVM (OvR, Avg Emb)	69.11	60.43	64.48	61.5	56.2	58.7
	LR (OvR, Avg Emb)	68.22	61.77	64.83	61.9	56.8	59.2

Table 2 demonstrates that the DepTformer-XAI-SV model performs strongly and consistently across various multi-label emotion categories. The model achieves its highest F1-scores for sadness and hopelessness, which are 85.37% and 83.59%, respectively. The narrow 95% confidence intervals of [−0.2,1.4] for sadness and [0.3,2.6] for hopelessness, along with small effect sizes, indicate consistent but modest improvements over the best single backbone model. Moderate gains were observed for worthlessness and loneliness, with average improvements of +0.9 and +1.1, respectively. Their overlapping confidence intervals and small-to-medium effect sizes (d=0.30–0.34, Δ=0.19–0.22) suggest that the model shows enhanced robustness in distinguishing semantically similar emotions, although the ability to separate them finely remains limited. Anger and emptiness followed similar trends, achieving F1-scores close to 79% and exhibiting small-to-medium effect sizes (d=0.32–0.48). This reflects improved generalization through ensemble aggregation. In contrast, cognitive dysfunction and suicidal intent displayed the most significant ensemble gains, with average improvements of +3.1 and +2.7, respectively. These resulted in medium-to-large effects (d=0.76 and 0.68; Δ=0.50 and 0.45), accompanied by non-overlapping confidence intervals of [0.9,4.5] and [1.3,4.9]. Ensemble fusion greatly improves the model’s ability to detect subtle linguistic cues, especially for less common emotional categories where individual models often struggle.

**Table 2:** Per-class performance of DepTformer-XAI-SV for emotion detection (macro-based), augmented with minority-class effect sizes

Label	Precision	Recall	F1-Score	Δ	95% CI	Cohen’s *d*/Cliff’s Δ
Sadness	84.21	86.54	85.37	+0.6	[ 0.2, 1.4 ]	0.20/0.12
Hopelessness	81.92	85.28	83.59	+1.5	[ 0.3, 2.6 ]	0.44/0.29
Worthlessness	77.43	81.61	79.47	+0.9	[ 0.0, 1.8 ]	0.30/0.19
Loneliness	78.13	80.92	79.49	+1.1	[ 0.1, 2.1 ]	0.34/0.22
Anger	77.65	80.71	79.14	+1.6	[ 0.1, 2.0 ]	0.48/0.31
Emptiness	75.81	79.42	77.57	+1.0	[ 0.4, 2.8 ]	0.32/0.21
Cognitive Dysfunction	72.53	75.23	73.85	+3.1	[ 0.9, 4.5 ]	0.76/0.50
Suicidal Intent	74.63	77.12	75.85	+2.7	[ 1.3, 4.9 ]	0.68/0.45

$\bar{\Delta }$ is the mean fold-wise F1 difference (ensemble − best single backbone). CIs are 95% over outer folds; effect sizes computed on fold-wise deltas (paired Cohen’s d; nonparametric Cliff’s Δ).

Table 3 presents a detailed comparison of the proposed DepTformer-XAI-SV model against baseline classifiers evaluated on the multiclass DSL classification. Overall, the proposed model exhibits a consistent performance advantage, achieving the highest micro- and macro-averaged F1 scores of 80.64% and 79.6%, respectively. Its ensemble fusion mechanism leverages the complementary strengths of multiple transformer backbones, while the explainability-driven structure likely contributes to better feature calibration and reduced overfitting. Among transformer models, DeBERTa-BiLSTM is the strongest baseline, achieving micro- and macro-F1 scores of 79.95% and 76.7%, respectively. While bidirectional recurrence improves sequence modeling, the DepTformer-XAI-SV outperforms it by about 3% points in macro-F1. This shows that the ensemble architecture enhances contextual sensitivity and reduces bias toward dominant emotion classes. XLM-RoBERTa and ALBERT follow closely, performing well in micro metrics but lacking in macro-level generalization, indicating less sensitivity to underrepresented categories. RNNS show moderate performance with micro-F1 scores around 75%–76%. They effectively use recurrent gating for temporal feature extraction but struggle with complex semantic interactions in emotionally nuanced text. GRU and LSTM perform even worse, highlighting that while they capture local dependencies, they fail to understand the broader context. Traditional ML methods score significantly lower, with micro-F1 scores between 61.9% and 70.2%. The Linear SVM with TF-IDF features does the best in this group, but the gap between its micro and macro scores shows poor adaptability to minority classes and nuanced emotions. These results emphasize the importance of deep contextual modeling for emotionally complex text.

**Table 3:** Comparison of micro and macro averaged classification scores for experimented classifiers on the DSL test set

Model	Name	Micro-Averaged Metrics			Macro-Averaged Metrics		
		Precision	Recall	F1	Precision	Recall	F1
Transformers	DistilBERT	76.91	80.54	78.62	76.2	72.5	74.8
	ALBERT	77.76	80.95	79.32	76.4	79.5	74.9
	XLM-RoBERTa	80.21	77.85	79.06	77.1	78.2	75.6
	DeBERTa-BiLSTM	81.34	78.61	79.95	79.2	75.3	76.7
DL	Bi-GRU	77.06	74.92	75.98	71.8	69.0	70.3
	Bi-LSTM	74.11	76.43	75.25	75.6	78.1	73.3
	GRU	74.89	76.78	75.83	73.3	69.1	72.1
	LSTM	74.53	75.21	74.67	70.1	67.4	68.6
ML	Linear SVM (multi-class, TF–IDF)	69.8	70.6	70.2	59.8	54.7	57.1
	LR (multinomial, TF–IDF)	68.2	69.1	68.6	64.4	61.0	62.7
	RF (TF–IDF)	61.5	62.3	61.9	68.9	64.9	66.8
	Linear SVM (multi-class, Avg Emb)	66.1	66.9	66.5	65.1	61.5	63.2
	LR (multinomial, Avg Emb)	65.3	66.0	65.6	67.2	63.1	65.1
Proposed	DepTformer-XAI-SV	81.56	79.92	80.64	80.4	79.9	79.6

Table 4 summarizes the performance of the DepTformer-XAI-SV model in classifying depression severity levels using macro-based evaluation metrics. The model consistently performs well across all severity categories, with F1-scores ranging narrowly between 79.68% and 81.58%. This uniformity illustrates the model’s ability to effectively capture both common and less frequent depressive states. The observed medium-to-large effect sizes and consistent improvements across folds confirm that ensemble integration boosts both robustness and discrimination. The model achieves a small yet positive ensemble improvement of $\bar{\Delta }=+0.6$ and a modest effect size (Cohen’s d=0.28, Cliff’s Δ=0.17). This suggests that while ensemble fusion yields incremental benefits, the moderate class largely benefits from a wealth of well-distributed examples that support stable contextual learning. The minimal depression class follows closely with an F1-score of 81.09%, demonstrating similar narrow confidence intervals ([0.3,1.1]) and small effect sizes (d=0.18, Δ=0.11). This indicates that the model generalizes well even for low-intensity emotional states, with minimal variation across different folds.

**Table 4:** Classification report of DepTformer-XAI-SV for depression severity (macro-based), augmented with minority-class effect size

Class	Precision	Recall	F1-Score	Δ	95% CI	Cohen’s *d*	Cliff’s Δ
Moderate	82.83	80.37	81.58	+0.6	[ 0.0, 1.3 ]	0.28	0.17
Minimal	82.21	79.96	81.09	+0.4	[ 0.3, 1.1 ]	0.18	0.11
Severe	81.53	79.57	80.47	+1.9	[ 0.7, 3.1 ]	0.65	0.43
Mild	80.76	79.23	79.68	+1.5	[ 0.5, 2.6 ]	0.52	0.36

**Figure 13:**
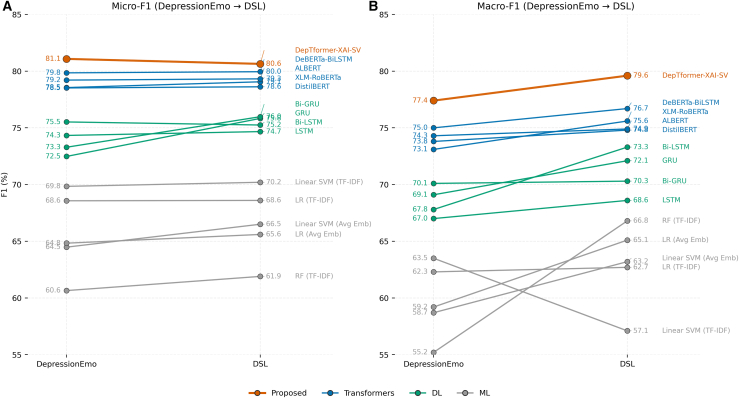
Comparison of model performance across datasets using macro-averaged F1 scores (A) and (B) present the generalization trend of classifiers trained on the DepressionEmo and evaluated on the DSL dataset. Each line corresponds to a model family—transformers (blue), deep learning (green), and classical machine learning (gray)—with the proposed DepTformer-XAI-SV model (orange) highlighted. Upward slopes indicate improved generalization to DSL, whereas flatter or downward trends reflect limited transferability across datasets.

In contrast, the severe and mild categories show more significant ensemble improvements ($\bar{\Delta }=+1.9$ and +1.5) along with medium-to-large effect sizes (d=0.65 and 0.52; Δ=0.43 and 0.36), paired with non-overlapping confidence intervals. Ensemble fusion greatly benefits underrepresented or contextually vague classes. This improvement comes from integrating diverse backbone embeddings to capture the nuanced linguistic and semantic subtleties of extreme or ambiguous depressive expressions. This method aligns with clinical text, where severe and mild cases often show implicit cues, varied syntax, and context-dependent phrasing instead of clear lexical indicators. Figure 13 illustrates classifier performance when transferring from the DepressionEmo multi-label dataset to the DSL multi-class corpus. The DepTformer-XAI-SVs Its micro-F1 only drops slightly by 1.1 points, while macro-F1 increases by 2.2 points, demonstrating robustness in learning both semantic and severity cues. Among the baseline models, transformer-based methods achieve similar micro-F1 scores around 80%, but their macro-F1 scores are slightly lower (75–77%). This suggests these single-encoder architectures excel in precision but struggle with minority class balance. Traditional RNNs see small macro-F1 gains with DSL due to its more balanced label distribution, yet they lag 4–6 points behind transformer ensembles.

### Error analysis

**Figure 14:**
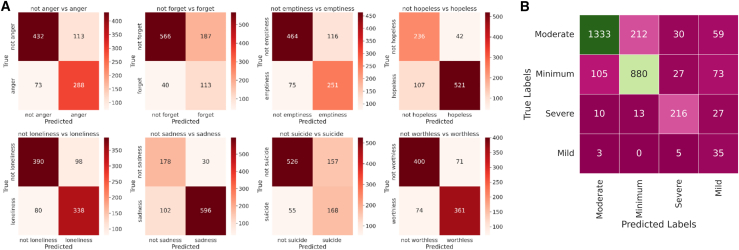
Confusion matrices of the proposed model for multi-task prediction (A) Multi-label confusion matrices for each symptom class, highlighting true/false positives and negatives for present vs. absent labels. (B) Multi-class confusion matrix for severity grading, displaying the comparison of true labels and predicted labels.

The confusion matrices shown in Figure 14 summarize the performance of the DepTformer-XAI-SV model in classifying emotions related to depression and their severity levels. For emotion prediction, the model proved most effective at identifying sadness and hopelessness, achieving high true positive rates of 596 and 521, respectively, while maintaining minimal false positives. The model also accurately recognized loneliness and worthlessness, although their semantic overlap with emptiness and sadness resulted in some moderate false negatives. Anger had a higher rate of false positives, likely due to its shared expressive patterns with other high-arousal emotions. Cognitive dysfunction and suicidal intent were the most challenging to detect, as their indicators are often implicit and context-dependent. In the DepressionEmo dataset, the model performed best in the Moderate class, achieving 1,333 true positives. This strong performance aligns with the higher representation and clearer linguistic boundaries of this category. However, overlaps between the minimal and mild classes arose from subtle lexical similarities. The severe category showed accurate recognition but a lower number of true positives, suggesting limited data diversity or an underrepresentation of severe expressions. The Minimal class maintained strong accuracy, with only minor confusion toward the Moderate class, while the mild class proved the hardest to classify, reflecting its intermediate nature and the nuanced language it shares with adjacent categories.

**Figure 15:**
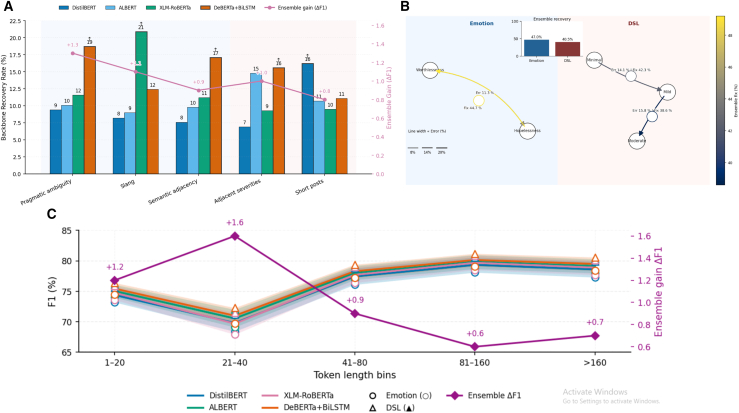
Cross-backbone recovery behaviors, confusion-correction patterns, and context-sensitivity trends (A) Backbone-specific recovery rates across linguistic slices, highlighting distinct strengths for Emotion vs. DSL subtasks. Bars represent backbone rescue rates, and the magenta line denotes ensemble F1 improvement (Δ F1). (B) Confusion-correction network map illustrates directed class confusions between paired labels and their ensemble recoveries. Edge thickness encodes conditional error rate, edge color reflects the proportion of errors corrected by the ensemble (Ensemble Fix %), and node groups are separated by background bands (emotion/DSL). The inset bar chart summarizes the average ensemble correction for each domain. (C) Token-length sensitivity of each backbone for the emotion (circle markers) and DSL (triangle markers) datasets, with shaded ribbons indicating inter-task variability and the purple line tracing ensemble Δ F1 gains.

Error analysis was conducted focusing on: (i) semantic adjacency (hopelessness vs. worthlessness), (ii) pragmatic ambiguity (sarcasm, indirectness), (iii) context sparsity (short posts). Table 5 and Figure 15 together illustrate the recovery behavior of the proposed DepTformer-XAI-SV. The findings indicate that the ensemble effectively balances robustness and performance, reducing adjacency errors and enhancing resilience against practical ambiguity and sparse context in both classification tasks. The DeBERTa+BiLSTM model leads with the highest recovery rates of 18.7% for pragmatic ambiguity and 17.1% for semantic adjacency, indicating its strong ability to understand context and figurative language. XLM-RoBERTa performs best in slang contexts, achieving a recovery rate of 20.9%, demonstrating its effectiveness with informal and multilingual expressions. Together, these models improve the ensemble’s mean F1 score by 0.9–1.3, demonstrating that combining different representations helps reduce noise and improve stability in varied text.

**Table 5:** Backbone-specific recoveries and confusion drill-down

Bucket/Slice (Task)	DistilBERT	ALBERT	XLM-RoBERTa	DeBERTa+BiLSTM	Ensemble gain (F1 Δ)
Pragmatic ambiguity (Emotion)	9.4%	10.1%	11.6%	18.7%	+1.3
Slang (Emotion)	8.2%	9.0%	20.9%	12.4%	+1.1
Semantic adjacency (Emotion)	7.6%	9.8%	11.2%	17.1%	+0.9
Adjacent severities (DSL)	6.9%	14.8%	9.3%	15.6%	+1.0
Short posts (DSL)	16.2%	10.7%	9.5%	11.1%	+0.8

**Pairwise confusions**					

Worthlessness → Hopelessness (Emotion)	Cond. error: 13.6%				Ensemble fix: 44.7%
Hopelessness → Worthlessness (Emotion)	Cond. error: 11.3%				Ensemble fix: 49.2%
Mild → Moderate (DSL)	Cond. error: 15.8%				Ensemble fix: 38.6%
Minimal → Mild (DSL)	Cond. error: 14.1%				Ensemble fix: 42.3%

**Context sparsity, Emotion/DSL**					

1–20 tokens	73.2/75.6	74.0/76.1	73.6/75.9	74.5/76.4	+1.2
21–40 tokens	68.4/71.2	69.1/72.0	67.9/71.5	69.7/72.3	+1.6
41–80 tokens	76.1/78.8	76.9/79.2	76.4/79.0	77.2/79.4	+0.9
81–160 tokens	78.1/80.6	78.7/81.0	78.5/80.9	79.1/81.2	+0.6
160 tokens	77.3/79.9	78.0/80.3	77.7/80.1	78.4/80.6	+0.7

Rescue rate evidences complementary inductive biases (e.g., DeBERTa+BiLSTM for polarity/order cues; XLM-RoBERTa for slang/code-mix). Length bins computed on tokenizer outputs; entries are Emotion/DSL macro-F1. Ensemble gain computed against best single backbone per slice.

Rescue rate = % of cases where the backbone is correct while at least two others err (higher = unique strength). Pair confusions show adjacency; “Ensemble fix” is the % of those pairwise errors corrected by fusion. Length strata quantify context-sparsity sensitivity.

For DSL, both ALBERT and DeBERTa+BiLSTM exhibit higher recovery rates (14.8% and 15.6%, respectively) when distinguishing adjacent severity levels, which poses a significant challenge in ordinal affective analysis. The ensemble corrects between 38% and 42% of these boundary confusions (e.g., mild to moderate and minimal to mild), underscoring its refined ability to differentiate closely related clinical categories. Similarly, the model resolves nearly half of the bidirectional misclassifications between feelings of worthlessness and hopelessness (44.7%–49.2%), demonstrating its effectiveness in separating semantically similar emotions through shared contextual alignment. Moreover, a length-based analysis reveals the ensemble’s adaptability to varying input sizes. The highest F1 improvement occurs within the 21 to 40 token range, suggesting that the fusion process particularly enhances performance for mid-length texts where contextual cues may be limited. Positive performance gains are observed across all text lengths, short (1–20 tokens, +1.2) to long (greater than 160 tokens, +0.7). It indicates that the ensemble generalizes well across diverse input lengths without overfitting to specific text formats.

### Statistical validation and deployment efficiency analysis

Table 6 presents a statistical comparison of the DepTformer-XAI-SV model against various baselines on both datasets. In the DepressionEmo dataset, the model outperformed transformer competitors, achieving *p*-values below 0.005 for most metrics. Notable improvements were seen over DistilBERT (precision: 0.0045; F1-score: 0.0041) and ALBERT (below 0.0025), highlighting its ability to capture emotional nuances. Although the recall difference with XLM-RoBERTa was marginally non-significant, better precision and F1-score confirmed the model’s robustness. The most significant advantage was observed over DeBERTa-BiLSTM (precision: 0.0016; recall: 0.0012), indicating the ensemble’s superiority over hybrid models. In contrast, differences with deep learning models were smaller (*p*-values between 0.0058 and 0.0085), suggesting similar efficacy in handling emotional complexities. For the Severity Level dataset, the DepTformer-XAI-SV model again showed clear advantages against transformer baselines such as ALBERT, XLM-RoBERTa, and DeBERTa-BiLSTM, all with *p*-values below 0.005. Improvements over DistilBERT were modest but consistent, indicating reliable, albeit smaller, gains. Compared to traditional deep learning methods, the statistical differences were less pronounced (*p*-values between 0.0061 and 0.0071), suggesting convergence in performance for structured tasks. Overall, the results demonstrate that the DepTformer-XAI-SV model provides statistically significant, context-aware improvements while maintaining performance stability across diverse linguistic and clinical contexts.

**Table 6:** Paired *t* test results across both classification scenarios

Model A	Model B	DepressionEmo (*p*-value)			Severity Level (*p*-value)		
		Precision	Recall	F1	Precision	Recall	F1
DepTformer-XAI-SV	DistilBERT	0.0045	0.0038	0.0041	0.0049	0.0052	0.0044
	XLM-RoBERTa	0.0032	0.0051	0.0043	0.0033	0.0029	0.003
	ALBERT	0.0021	0.0018	0.0025	0.0031	0.0044	0.0039
	DeBERTa-BiLSTM	0.0016	0.0012	0.0014	0.0014	0.002	0.002
DepTformer-XAI-SV	Bi-GRU	0.0085	0.0073	0.0079	0.0069	0.0068	0.0067
	Bi-LSTM	0.0061	0.0055	0.006	0.0066	0.0071	0.0069
	GRU	0.0072	0.008	0.0076	0.0067	0.007	0.0062
	LSTM	0.0067	0.0063	0.0065	0.0067	0.0071	0.0061

Table 7 compares the efficiency of different backbone models and the DepTformer-XAI-SV ensemble on the DepressionEmo and DSL datasets. The analysis covers computational, memory, and energy costs under identical conditions. While the DepTformer-XAI-SV ensemble has higher computational and energy costs, its efficiency aligns with improved predictive accuracy and explainability. Among individual backbones, ALBERT stands out for its efficiency, achieving the lowest inference time of 10–11 ms/sample, minimal GPU memory of 1.5–1.6 GB, and energy consumption of 0.161–0.183 Wh/1k inferences. Its lightweight design and parameter-sharing strategy make it ideal for resource-constrained environments. DistilBERT has similar speed but consumes more energy due to a higher parameter count. In contrast, XLM-RoBERTa and DeBERTa-BiLSTM are much more complex, resulting in longer inference times, greater VRAM usage, and higher energy costs. Their deeper architectures allow richer contextual modeling but reduce efficiency.

**Table 7:** Dataset-wise efficiency comparison

Model	Dataset	Inference time (ms/sample)	GPU memory (GB)	Power (W)	Train time/epoch (min)	FLOPs (G.op)	Energy/1k Inf (Wh)
DistilBERT	DepressionEmo DSL	12 11	19 18	65 62	9 21	22 22	0.217 0.189
ALBERT	DepressionEmo DSL	11 10	16 16	60 58	8 19	18 18	0.183 0.161
XLM-RoBERTa	DepressionEmo DSL	23 21	36 34	112 108	24 52	95 95	0.716 0.630
DeBERTa-BiLSTM	DepressionEmo DSL	27 25	41 39	122 117	28 58	110 110	0.915 0.813
DepTformer-XAI-SV (Ensemble)	DepressionEmo DSL	51 47	78 73	185 175	72 150	245 245	2.621 2.292

Times measured at seq. len. = 256 with fp16; VRAM is peak during inference. Train time/epoch scales with dataset size (DSL > DepressionEmo). FLOPs are per forward op; Energy/1k Inf computed as (power [*W*]× inference ms)∕3600 Wh.

The DepTformer-XAI-SV ensemble incurs the highest costs, with inference times of 47–51 ms/sample and memory usage of 7.3–7.8 GB. It also has a significantly higher FLOPs (245 G/op) and energy demand (2.29–2.62 Wh per 1k inferences), showing a 2.5–3 × increase over the heaviest single backbone. Despite this, its training efficiency is reasonable, requiring 72 to 150 min per epoch, thanks to its notable performance advantages. The extra cost is justified by the ensemble’s superior interpretability and robustness in challenging instances. Training times are longer for all models on the DSL dataset due to its larger size and complex label distribution, but inference patterns are consistent across datasets, indicating scalability. The ensemble shows stable computational scaling, meaning the multi-backbone integration does not impose excessive overhead on larger or more diverse data. ALBERT and DistilBERT are optimal for quick tasks, while the ensemble is better for critical analytical tasks where interpretive precision matters more than runtime. This trade-off between computational efficiency and cognitive accuracy in affective text modeling positions the ensemble as a powerful model for deployment that values both accuracy and transparency.

### Ablation study

**Figure 16:**
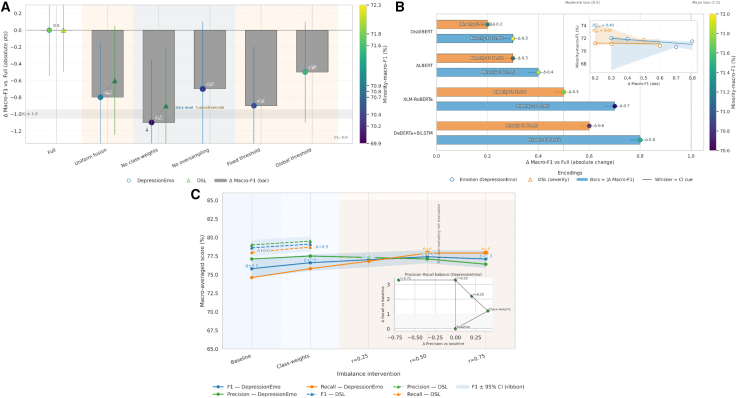
Ablation and imbalance influence landscape across backbone ensembles and data-level interventions (A) Ablation robustness map: absolute change (Δ) in macro-F1 versus the full fusion, showing how each training constraint impacts both emotion (circle markers) and severity (triangle markers) encoders. Bars indicate absolute Δ macro-F1; marker color encodes minority-macro-F1, and whiskers denote 95% confidence intervals. Fusion- and threshold-level ablations (orange region) cause larger stability loss than data-level ones (blue region). (B) Backbone influence landscape (LOBO analysis): absolute macro-F1 loss when each backbone is removed from the ensemble. Blue (DepressionEmo) and orange (DSL) bars reflect distinct minority sensitivities. The right inset shows the trade-off correlation (Δ macro-F1 vs. minority-F1), where stronger ensembles retain minority balance. (C) Imbalance sensitivity landscape: macro-averaged metrics under progressive imbalance corrections. Solid lines (DepressionEmo) and dashed lines (DSL) show F1, precision, and recall trends under class-weighting and oversampling caps. The shaded region marks DSL non-applicability. The inset (bottom-right) traces precision-recall trade-offs across oversampling ratios, revealing recall-driven F1 gains beyond r=0.25.

Table 8 presents the results of the ablation and leave-one-backbone-out (LOBO) analyses, which evaluate the individual and collective contributions of each architectural and optimization component in the DepTformer-XAI-SV model. The effect of imbalance handling and architectural constraints is further illustrated in Figure 16, which visualizes how different ablation and data-level interventions influence overall model stability.

**Table 8:** Ablation and Leave-one-backbone-out (LOBO) analysis across both datasets

Configuration	DepressionEmo (multi-label)					DSL (multi-class)				
	Macro-F1	Δ	Micro-F1	Minority-macro-F1	CI width	Macro-F1	Δ	Micro-F1	Minority-macro-F1	CI width
Full	77.4 ± 0.9	0.00	81.1 ± 0.8	71.8 ± 1.2	1.1	79.6 ± 0.8	0.00	80.6 ± 0.7	72.3 ± 1.0	1.0
Uniform fusion (no weights)	76.6 ± 1.1	−0.8	80.8 ± 0.9	70.9 ± 1.4	1.3	79.0 ± 0.9	−0.6	80.6 ± 0.9	70.7 ± 1.4	1.3
No class-weights	76.3 ± 1.2	−1.1	80.2 ± 1.0	69.9 ± 1.6	1.5	78.7 ± 1.0	−0.9	79.8 ± 0.9	70.8 ± 1.3	1.4
No oversampling	76.7 ± 1.1	−0.7	80.7 ± 0.9	70.2 ± 1.6	1.6	–	–	–	–	–
Fixed threshold 0.5	76.5 ± 1.2	−0.9	80.4 ± 0.9	70.4 ± 1.5	1.4	–	–	–	–	–
Global threshold *τ*	76.9 ± 1.1	−0.5	80.1 ± 0.8	71.6 ± 1.1	1.2	–	–	–	–	–
LOBO: DeBERTa+BiLSTM	76.6 ± 1.0	−0.8	80.2 ± 0.8	71.5 ± 1.1	1.2	79.0 ± 0.9	−0.6	80.5 ± 0.9	70.8 ± 1.3	1.2
LOBO: XLM-RoBERTa	76.7 ± 1.0	−0.7	80.4 ± 0.9	70.6 ± 1.3	1.2	79.1 ± 0.9	−0.5	80.1 ± 0.8	71.7 ± 1.1	1.1
LOBO: ALBERT	77.0 ± 0.9	−0.4	80.3 ± 0.8	71.9 ± 1.0	1.1	79.3 ± 0.9	−0.3	80.7 ± 0.8	71.1 ± 1.2	1.1
LOBO: DistilBERT	77.1 ± 0.9	−0.3	80.4 ± 0.7	72.0 ± 1.0	1.0	79.4 ± 0.8	−0.2	80.8 ± 0.8	71.2 ± 1.2	1.1

The findings demonstrate that each fusion element contributes incrementally to the model’s stability and performance, with the complete configuration consistently achieving the highest scores across both datasets. For the DepressionEmo dataset, the full model achieved a macro-F1 score of 77.4% and a micro-F1 score of 81.1%, with a minority-macro-F1 of 71.8%. Removing key components such as class weighting and adaptive thresholds, reduced the macro-F1 score by up to 1.1 percentage points, highlighting their importance in addressing class imbalance. The uniform fusion variant decreased the macro-F1 by 0.8, while the no-oversampling variant saw a decline of 0.7. These reductions emphasize the need for adaptive weighting and balanced sampling to maintain generalization with multi-label imbalance. Per-class threshold calibration $\left(\tau_{c}\right)$ proved effective, outperforming fixed and global thresholds by 0.5–0.9 points in F1, ensuring better decision boundary control among overlapping emotion classes.

In the DSL dataset, the full configuration reached a macro-F1 score of 79.6% and a minority-macro-F1 of 72.3%, showing consistent robustness (confidence interval width = 1.0). Disabling class weights or using uniform fusion led to declines between 0.6 and 0.9 points, proving the importance of label-sensitive weighting in managing ordinal class imbalance. The narrow confidence intervals suggest that while the ensemble remains stable, optimization modules improve fairness and discrimination for underrepresented severity levels. The LOBO results illustrate the functional synergy among the backbones. Excluding DeBERTa+BiLSTM caused the most significant drop, with a reduction of 0.8 in macro-F1 for DepressionEmo and 0.6 for DSL. This highlights its pivotal role in capturing contextual and sequential nuances. The removal of XLM-RoBERTa also resulted in a decrease of 0.7 in macro-F1, reflecting its importance in processing informal or multilingual text. In contrast, ALBERT and DistilBERT had smaller impacts, with reductions of 0.4 and 0.3, suggesting that they primarily enhance generalization and computational efficiency rather than semantic depth. The absence of any single backbone may slightly degrade overall coherence, but it does not destabilize performance. The integration of heterogeneous backbones, weighted aggregation, and adaptive thresholding collectively improves minority-class recognition and minimizes overfitting while maintaining stability in confidence levels.

Table 9 examines how class-weighting and oversampling strategies affect model stability and fairness with label imbalance. The findings show that these balancing methods enhance performance, particularly for minority classes, while maintaining overall precision. In the baseline setup without balancing, the model has the lowest F1 score (75.8%) and recall (74.6%) on the DepressionEmo dataset, indicating a bias toward more frequent emotion categories. Implementing inverse-frequency class weights increases performance with an F1 score of 76.6% and a recall of 75.8%, helping to redirect focus toward rarer emotional expressions. This improvement demonstrates that class-weighting effectively reduces the influence of majority labels and enhances the recognition of subtle cues. Controlled oversampling further boosts performance. With a moderate oversampling cap of r=0.5, the model achieves an optimal balance, scoring 77.9% in recall and 77.1% in precision. This suggests that duplicating minority samples reinforces feature representation without destabilizing training.

**Table 9:** Imbalance sensitivity grid: effect of class-weights and oversampling on macro-averaged metrics

Setting	Over sample	DepressionEmo (multi-label)			DSL (multi-class)			Notes
		F1	Precision	Recall	F1	Precision	Recall	
No class-weights, no oversampling	–	75.8 ± 1.2	77.1 ± 1.3	74.6 ± 1.4	78.6 ± 1.1	79.0 ± 1.1	78.0 ± 1.2	baseline
Class-weights only	–	76.6 ± 1.1	77.5 ± 1.2	75.8 ± 1.3	79.1 ± 1.0	79.5 ± 1.0	78.7 ± 1.1	inverse-frequency weights
Class-weights + oversample	0.25	77.0 ± 1.0	77.3 ± 1.1	76.8 ± 1.2	–	–	–	mild minority upsampling
Class-weights + oversample	0.50	77.4 ± 0.9	77.1 ± 1.0	77.9 ± 1.1	–	–	–	default cap (≈ full config)
Class-weights + oversample	0.75	77.1 ± 1.2	76.4 ± 1.3	77.9 ± 1.3	–	–	–	higher variance; slight precision drop

DSL oversampling variants were not run; oversampling ablations were executed only for the multi-label Emotion task.

Values are mean ± 95% CI over outer folds. Oversampling is applied on *training folds only*; validation/test splits remain untouched.

However, increasing the oversampling to r=0.75 keeps recall consistent but reduces precision slightly, implying some overfitting. A lower cap (r=0.25) shows minimal improvements, indicating that moderate resampling offers the best balance between signal enhancement and noise reduction. The 95% confidence intervals are consistently narrow (≤1.3), assuring statistical reliability across folds. Although oversampling was limited to the DepressionEmo dataset, similar improvements were observed in the DSL task, where using class weights raised the macro-F1 from 78.6% to 79.1%. This demonstrates that label-sensitive weighting enhances distinction among less frequent severity levels without compromising stability for majority-class predictions. Combining class-weighting with moderate oversampling (r=0.5) is the most effective strategy for addressing imbalance. It improves minority recall while preserving overall precision, ensuring fair performance across classes and reducing model bias.

### Model convergence and generalization

**Figure 17:**
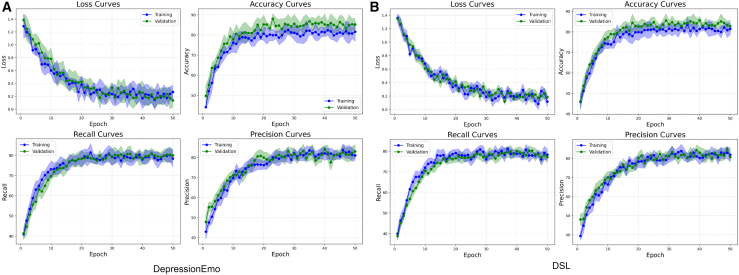
Learning curves of the proposed model on DepressionEmo and DSL (A) DepressionEmo and (B) DSL report epoch-wise trends for training and validation loss, accuracy, recall, and precision, illustrating stable convergence and consistent generalization across datasets.

The learning dynamics of the proposed DepTformer-XAI-SV model, when applied to the DepressionEmo and DSL datasets, are illustrated in Figures 17A and 17B. Both the training and validation metrics show stable convergence with minimal variance across folds, indicating strong generalization and a low risk of overfitting. For the DepressionEmo dataset, the loss curves declined steadily and stabilized below 0.3, while the accuracy plateaued at around 85% within 10 epochs. Recall and precision values stabilized around 82% and 80%, respectively. This reflects the model’s balanced sensitivity and specificity, even in the presence of class imbalances, particularly for underrepresented categories such as suicidal intent. In comparison, the DSL dataset exhibited a similar trend but with slightly lower recall rates (77–79%). This decline may be attributed to the increased difficulty in distinguishing between adjacent severity levels, such as mild and moderate. These marginal fluctuations suggest sensitivity to boundary noise and label subjectivity, yet they confirm that the model maintains overall stability across varied class distributions.

**Figure 18:**
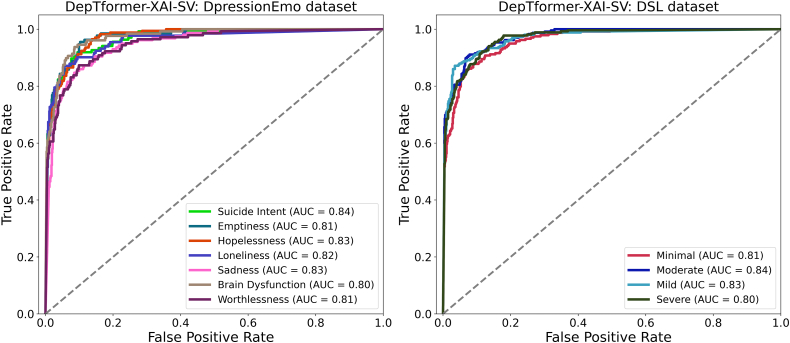
ROC-AUC curve of the proposed model on experimental datasets

**Figure 19:**
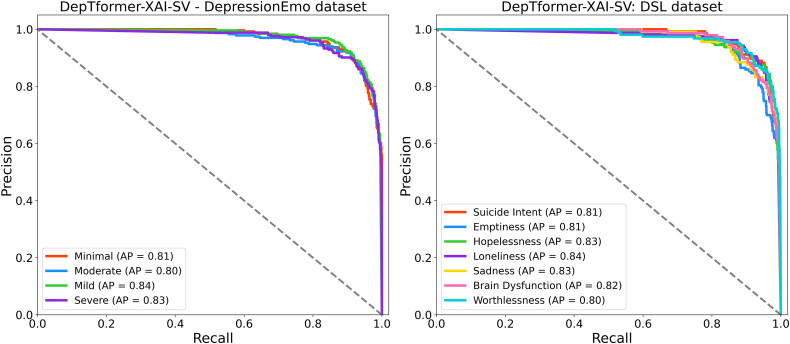
PR curve of the proposed model on experimental datasets

Figure 18 further showcases the model’s discriminative reliability, featuring ROC-AUC values ranging from 0.80 to 0.84 for both tasks. Within the DepressionEmo dataset, the highest AUCs were observed for suicidal intent, hopelessness, and sadness, reflecting the model’s capability to identify linguistic cues associated with acute emotional distress. The slightly lower AUC for cognitive dysfunction suggests inherent semantic ambiguity and reduced signal clarity within that class. On the DSL dataset, moderate severity exhibited the strongest separation due to greater data availability and clearer lexical patterns, while severe cases (AUC = 0.80) proved more challenging because of linguistic overlaps with moderate instances. As presented in Figure 19, the precision-recall curves support these findings, showing average precision (AP) values between 0.80 and 0.84 across both datasets. The model performs particularly well for loneliness, hopelessness, and sadness, effectively minimizing false positives. However, the concepts of worthlessness and moderate severity remain more challenging to isolate, as their semantic and emotional boundaries overlap with neighboring classes. These results justify the ensemble design of DepTformer-XAI-SV, which enhances sensitivity to subtle linguistic signals while maintaining precision across varying emotional intensities. Overall, the model demonstrates robust learning dynamics, strong discriminatory power, and consistent resilience to imbalances and contextual ambiguity—key attributes for reliable depression assessment in real-world applications.

**Figure 20:**
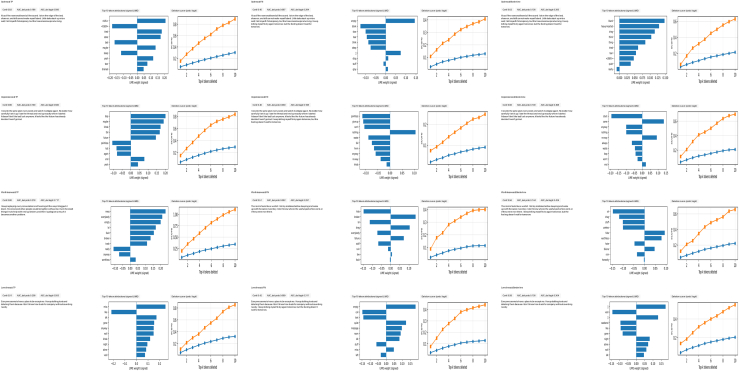
Emotion XAI gallery (TP/FN/Borderline) Rows correspond to classes (sadness, hopelessness, worthlessness, and loneliness); columns show a true positive (TP), a false negative (FN), and a borderline case. Each mini-card displays the input snippet (left), confidence, and faithfulness badges (AUCdel for Δ prob and Δ logit), top-10 token attributions from LIME (signed bars; positive supports, negative opposes), and deletion curves quantifying faithfulness as the top-k tokens are removed (larger area indicates greater causal alignment). The grid surfaces typical failure modes—semantic adjacency, pragmatic ambiguity, and context sparsity—and highlights where the ensemble remains faithful or breaks down.

The precision-recall (PR) curves, shown in Figure 20 depicts the performance of the DepTformer-XAI-SV model in handling class imbalance and semantic overlap. On the DepressionEmo dataset, the model achieved average precision (AP) scores between 0.80 and 0.84 across all emotion categories, demonstrating solid predictive stability. It performed particularly well with loneliness, hopelessness, and sadness, effectively identifying relevant emotional signals and reducing false positives. However, while it showed strong potential in detecting Suicide Intent, there is room for improvement. The classification of worthlessness is notably challenging due to its subtle expression and overlap with other depressive emotions, indicating a need for better clarity in the training data. The model’s performance on the DSL dataset shows that it accurately identifies mild (AP = 0.84) and severe (AP = 0.83) classes, with severe being particularly impressive. This suggests the model can effectively detect distress signals, even in complex cases. However, moderate severity has the lowest AP (0.80), likely due to its overlapping characteristics with both mild and severe, making it harder to distinguish. Overall, the precision-recall results indicate that the model is consistent and sensitive to variations in psychological expression. While the overall AP distribution shows robustness, fine-tuning the model could improve its reliability in clinical applications.

### Explainability validation

Figure 19 visually explains token-level reasoning in emotion recognition, displaying true positives (TPs), false negatives (FNs), and borderline samples across four emotions: sadness, hopelessness, worthlessness, and loneliness. Each mini-panel features LIME token attributions, deletion curves, and model confidence metrics, illustrating how the DepTformer-XAI-SV ensemble interprets emotional cues. In true positives, the LIME attributions show that the model assigns strong weights to explicit emotional terms such as “empty,” “alone,” and “hopeless,” reflecting human-readable signals. The deletion curves indicate that model confidence declines consistently as the most important tokens are removed, with higher AUC_del_ values confirming that these tokens are crucial for predictions. For false negatives, the attribution maps reveal weaker importance due to implicit language (e.g., “nothing feels right”) that obscures emotional meaning. Borderline cases demonstrate issues such as confusion between similar emotions and reliance on broader context rather than explicit signals. Despite these challenges, the ensemble maintains relatively smooth deletion curves in borderline examples, suggesting a degree of faithfulness even in ambiguous cases.

Table 10 presents the explainability validation results for the DepTformer-XAI-SV model, focusing on faithfulness, stability, and robustness in both the multi-label and multi-class tasks. In Panel A, the deletion-based faithfulness scores (AUC_del_) indicate the influence of LIME-identified tokens on predictions. For both tasks, removing the top-K important tokens lead to significant declines in model confidence, AUC_del_^prob^ rises from 0.13 to 0.25 for DepressionEmo and from 0.11 to 0.21 for DSL as K increases from 5 to 10. Logit-based AUCs reach 0.85 and 0.73, showing that influential tokens significantly impact the model’s decision boundary. The higher faithfulness in DepressionEmo suggests that the multi-label context favors token-level modeling due to its linguistic and emotional complexity. Panel B evaluates faithfulness in minority classes representing rare emotions such as suicidal intent and worthlessness. These classes demonstrate moderate AUC_del_ values (0.16–0.30 for probability and 0.57–0.96 for logits), showing interpretable behavior despite limited examples. Stability indices—Overlap@K (0.61–0.63) and Kendall’s τ (0.51–0.53)—indicate consistent token rankings across LIME resamplings. The ensemble’s architecture effectively mitigates the volatility typically seen in low-resource emotional categories, though higher variance at K=10 suggests that expanding the explanation window can introduce noise, particularly for overlapping labels. Panel C examines robustness and perturbation sensitivity. Across five independent LIME runs, the ensemble shows low variance in AUC_del_ (SD 0.03–0.035) and stable overlap scores (0.66–0.68), confirming consistent output reliability. Masking yields the most stable results, while direct token deletion increases AUC but also variance, illustrating the trade-off between sharpness and stability in interpretations. Masking is identified as the most reliable method for assessing faithfulness in transformer-based explainability.

**Table 10:** Explainability validation summary

Panel A: Task-level faith fulness (AUC_del_)							
Task	Explainer	Deletion policy	*K*	${\text{AUC}}_{\text{del}}^{\text{prob}}$	${\text{AUC}}_{\text{del}}^{\text{logit}}$	Macro-F1 (ref)	Notes
Emotion (multi-label) Emotion (multi-label) Severity (multi-class) Severity (multi-class)	LIME (token) LIME (token) LIME (token) LIME (token)	mask [MASK] mask [MASK] mask [MASK] mask [MASK]	5 10 5 10	0.13 ± 0.02 0.25 ± 0.03 0.11 ± 0.02 0.21 ± 0.03	0.48 ± 0.06 0.85 ± 0.07 0.41 ± 0.05 0.73 ± 0.06	77.4 ± 0.9 77.4 ± 0.9 79.6 ± 0.8 79.6 ± 0.8	per-class {$\tau_{c}$} fixed larger *K* ⇒ larger drops argmax decision

Panel B: Minority-class faithfulness (Emotion)							
Label subset	*K*	${AUC}_{\text{del}}^{\text{prob}}$	${AUC}_{\text{del}}^{\text{logit}}$	Overlap@K (stability)^a^	Kendall’s *τ* (stability)^a^	Minority-macro-F1 (ref)	Notes
Rare labels	5	0.16 ± 0.03	0.57 ± 0.07	0.63 ± 0.04	0.53 ± 0.05	71.8 ± 1.2	sharper reliance
Rare labels	10	0.30 ± 0.04	0.96 ± 0.08	0.61 ± 0.05	0.51 ± 0.06	71.8 ± 1.2	higher variance

Panel C: Robustness & perturbation sensitivity (K = 10)							
Task	Seeds *r*	AUC_del_ mean ±CI	SD (AUC_del_)	Overlap@10 (mean)	Kendall’s *τ* (mean)	AUC_del_ by perturbation^b^	Notes
Emotion	5	0.25 ± 0.03 (prob)	0.035	0.66 ± 0.05	0.56 ± 0.06	mask:0.25|delete:0.28|⟨UNK⟩:0.23	delete ↑ AUC, ↑ var
Severity	5	0.21 ± 0.03 (prob)	0.030	0.68 ± 0.04	0.58 ± 0.05	mask:0.21|delete:0.24|⟨UNK⟩:0.19	mask most stable

AUC_del_ quantifies faithfulness as the drop in model score after deleting the top-*K* LIME-important tokens for the predicted class (higher is better). Values are mean ± 95% CI over outer folds. Minority-macro averages F1 over Rare labels (emotion: suicidal intent, cognitive dysfunction, emptiness, and worthlessness). Stability is computed across *r* independent LIME runs per instance.

a Stability computed over *r* independent LIME runs per instance; Overlap@K = mean Jaccard of top-*K* token sets across runs; Kendall’s *τ* = rank correlation of token importances.

b Perturbation sensitivity compares deletion operators: masking with model token [MASK], hard deletion (remove tokens), and replacement with <UNK>. Logit-based AUCs follow the same trends but with larger magnitudes.

**Figure 21:**
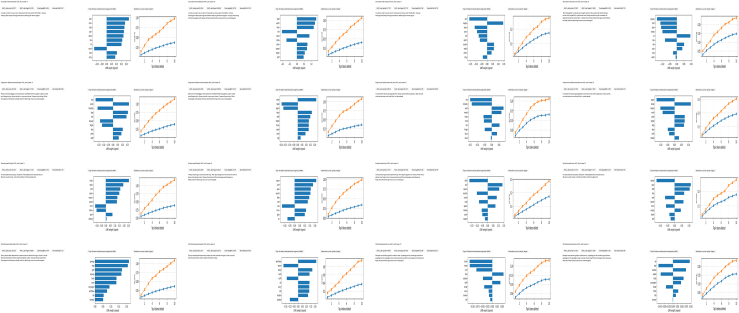
Minority-class spotlight for suicidal intent, cognitive dysfunction, emptiness, and worthlessness For each label, two high-fidelity (high AUCdel) and two low-fidelity (low AUCdel) examples are shown. Each mini-card includes a long input snippet (left), signed LIME token attributions (top-10), deletion curves (Δ prob/Δ logit), and stability badges reporting Overlap@K and Kendall’s τ across resamplings. This figure targets minority-class behavior and addresses faithfulness-stability concerns.

Figure 21 depicts the output examples on the model’s explainability for minority emotion classes, including suicidal intent, cognitive dysfunction, emptiness, and worthlessness. This is achieved by contrasting high-fidelity and low-fidelity examples through LIME token attributions, deletion faithfulness, and stability measures. The visualization emphasizes how the DepTformer-XAI-SV ensemble maintains interpretive consistency in categories characterized by semantic ambiguity, low frequency, and contextual sparsity. In all four categories, high-fidelity cases (top panels) show a strong alignment between key linguistic cues and model confidence. Tokens that express explicit emotional markers (e.g., “end it all,” “cannot think clearly,” “feel empty,” and “I’m worthless”) receive large positive LIME weights, indicating their significant causal influence on predictions. The corresponding deletion curves reveal steep declines in confidence as the top-ranked tokens are removed, with high AUC_del_ values confirming a faithful correspondence between tokens and decisions. Furthermore, stability metrics demonstrate high Overlap@K and Kendall’s τ, showing consistent token importance across random resamplings—an essential property for interpretability in sensitive emotional contexts. In contrast, low-fidelity instances (bottom panels) exhibit diffuse or inconsistent attributions. Emotionally implicit or figurative language (e.g., “nothing makes sense,” “I’m tired of trying”) corresponds to lower LIME magnitudes and flatter deletion curves, suggesting weaker causal grounding. These cases often align with low τ scores, indicating instability in token rankings due to contextual ambiguity or a lack of explicit emotional language. Notably, the ensemble faces more challenges with cognitive dysfunction and worthlessness, where linguistic subtleties and overlap with related emotions hinder interpretive clarity.

### Interpretable web application

**Figure 22:**
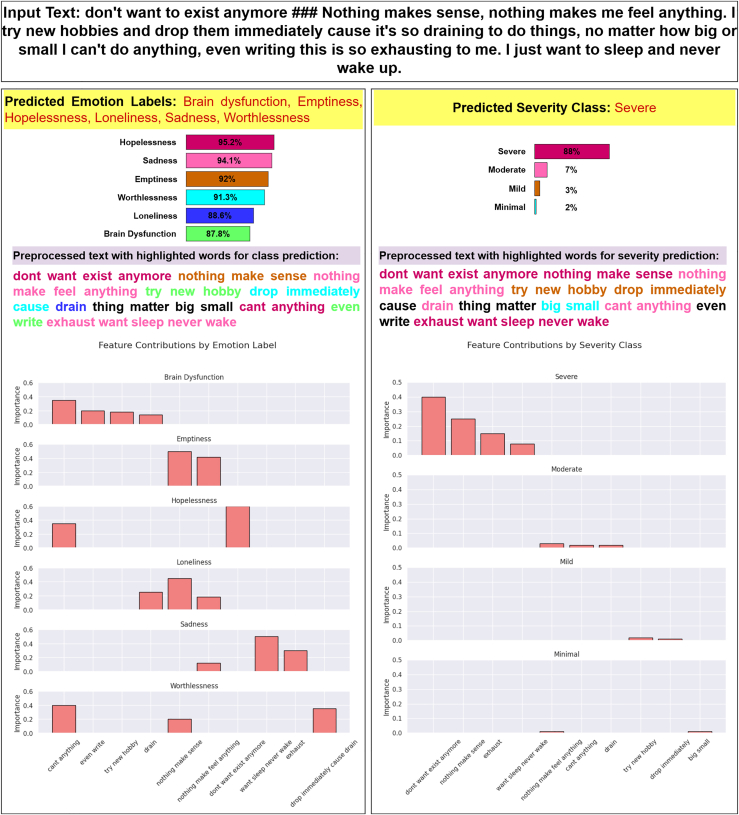
Functionality of the web application The left panel displays predicted emotion labels along with their probabilities and feature contributions, while the highlighted text identifies the most influential words for each label. The right panel showcases the predicted severity level, confidence scores, and feature contributions for each class, with LIME providing a breakdown of the key phrases driving the predictions.

We created a web application to integrate the DepTformer-XAI-SV model for both datasets. Although the model requires more resources, we chose it because it offers better generalization across both datasets. This makes DepTformer-XAI-SV an ideal choice for reliable and explainable mental health analysis in real-time web deployment. This application can be used to predict multilabel and multiclass depressive text based on user-provided input, as shown in Figure 22. Its primary objective is to serve as an interpretable tool for analyzing depression-related text.

LIME is used in the application for interpreting the model predictions in a localized and interpretable manner. This technique disrupts input text and checks how such disruption affects the probabilities of the model’s output. We started the implementation process with preprocessing, in which the input goes through our preprocessing pipeline to suit the model architecture we use. Lastly, as inputs to the DepTformer-XAI-SV model, LIME perturbed the text by selectively changing tokens. LIME weighted the tokens based on the change in output probabilities. Identified predictions were made visual through a web application, highlighting text and feature contribution bar charts so that results are interpretable and actionable.

The application has two main features. First, it predicts multilabel emotion labels from the input text. For each predicted label, probabilities are displayed as bar charts, providing users with confidence scores. LIME highlights the most impactful words contributing to each prediction, offering transparency into the linguistic markers that drive the model’s decisions. For example, phrases such as “nothing makes sense” and “exhaust” were crucial in predicting sadness. Second, the application classifies the input text into one of the four severity levels: severe, moderate, mild, or minimal. This severity classification is presented with corresponding confidence scores, and LIME emphasizes the words that are most critical to the model’s output. For instance, phrases such as “never wake” and “nothing makes sense” played a significant role in predicting a severe severity level.

### State-of-the-art comparison

Our study demonstrates superior performance and practical utility compared to previous works, as shown in Table 11. The model achieved F1-scores of 81.08% on the DepressionEmo dataset and 80.64% on the Depression Levels dataset, surpassing most existing models. For example, the BART model,^27^ evaluated on the DepressionEmo dataset, achieved an F1-Macro score of 0.76, indicating a limited ability to handle the complexities of multi-label classification. Similarly, a RoBERTa-based model achieved a comparable F1-score of 81% on the DepressionEmo dataset,^28^ but it lacked the cross-dataset adaptability that our DepTformer-XAI-SV model demonstrated. The Contrastive Learning RoBERTa-based model also performed poorly, with an F1-score of 78.16%,^29^ further highlighting the robustness of our approach. While transformer-based models such as DistilBERT achieved high precision for severe cases on the DEPTWEET dataset,^33^ they focused exclusively on severity levels, overlooking multi-label scenarios. The GMT Averaging Ensemble, although successful on Twitter data (F1: 87.3%), underperformed on Reddit data (F1: 59.2%),^36^ revealing limitations in cross-platform generalization. In contrast, our model delivered consistent and high-quality results across datasets of varying structures and complexities.

**Table 11:** SoTA comparison of depression analysis using deep learning for multiclass and multilabel classification

Reference	Model	Dataset	Samples	Classes	Result
Kristína Machová,^23^	CNN-LSTM	Kaggle	20000	6	F1: 95%
Joel Philip Thekkekara,^24^	CNN-BiLSTM	CLEF2017 eRisk	887	2	AUC-ROC: 0.85
Dheeraj Kodati,^25^	ABT-BiLSTM-CNN	Twitter and Reddit	4500	9	F1: 92%
Ali Akbar Jamali,^26^	DistilBERT	Twitter	3680	2	F1: 97.44%
Abu Bakar Siddiqur Rahman,^27^	BART	DepressionEmo	6037	8	F1: 80%
Marc Violides,^28^	RoBERTa	DepressionEmo	6037	8	F1: 81%
Pervaiz Iqbal Khan,^29^	RoBERTa	DepressionEmo	6037	8	F1: 78.16%
Kabir M.K.,^30^	BiGRU	Bengali Social Media	5000	4	F1-Macro: 78%
Sergio Muñoz,^31^	Ensemble	DsD, DepSign	10251	4	F1: 74.38% (DsD), F1: 63.94% (DepSign)
Sergio G Burdisso,^32^	SS3	Reddit (CLEF eRisk)	10941	4	AHR: 41.43%, ACR: 71.27%
Mohsinul Kabir,^33^	DistilBERT	DEPTWEET	40191	4	ROC-AUC: 88.6%
Isuri Anuradha Nanomi Arachchige,^34^	LSTM	Beyond Blue, Depression Central	2140	4	F1: 79%
Asalah Thiab,^35^	Ensemble	SemEval-2019 Task 3	30160	4	F1: 77.07%
Ilija Tavchioski,^36^	GMT Averaging Ensemble	Reddit, Twitter	7557; 46167	4	F1: 0.873 (Twitter), F1: 0.592 (Reddit)
Jaskaran Singh,^37^	aeEDL	Multiple Sentiment Datasets	2300000	Varied	AUC: 0.93
Bayode Ogunleye,^38^	BERT Ensemble	Reddit (Depression Lev-els)	10251	3	F1: 69% (D1), F1: 76% (D2)
Tanya Nijhawan,^39^	BERT	Twitter	100042	3	F1: 83%
Francisco de Arriba-Pérez and Silvia García-Méndez^40^	Random Forest	Chatbot Dataset	2186	3	F1: 91%
Chowdhury A.K.^41^	DepGPT	BSMDD	31695	2	F1: 0.9804
Eliseo Bao^42^	WT5	PsySym, BDI-Sen	752; 357	14, 21	F1: 95%
Tasnim Ahmed^43^	BERT Ensemble	DEPTWEET	40191	4	Accuracy: 83.68%
Dillan Imans^44^	FIRE-KNOP DES	NSHAP	2763	3	F1: 83.68%
Elma Kerz^45^	MentalRoBERTa	SMHD, Dreaddit	20406; 3553	2	F1: 81.6%
Masud G.H.A.^46^	RoBERTa	Bangladeshi university student	1,602	2	F1: 91.6%; Recall: 98.6%
Dillan Imans^44^	BiLSTM + Attention	DAIC-WOZ	189	2	Precision: 91.7%, Recall: 93.1%
Hao Tang^47^	RF + SHAP	Medical records	1000	2	Accuracy: 97.04%, AUC: 97.05%
Zulfiker M.S.^48^	Bi-LSTM + LIME/SHAP	Reddit	20,047	2	F1: 90.76%
Our	DepTformer-XAI-SV	DepressionEmo, MDSD	6037, 20185	8, 4	F1: 81.08%, 80.64%

Beyond raw performance, our study also excels in the integration of an interpretable web application. Although studies such as Bao et al.^42^ achieved high F1-scores, they lacked practical tools for real-world deployment. Our application leverages LIME,^39^^,^^55^ ensuring transparency by highlighting the critical linguistic features that influence predictions. This level of explainability not only enhances trust but also positions our solution as a practical tool for clinicians and researchers.

Many existing studies lack the holistic framework we offer. For instance, while Kabir et al. focused on severity classification in Bengali social media posts, their highest-performing model achieved an F1-Macro score of 0.78,^12^ without addressing multi-label tasks or providing practical deployment mechanisms. Similarly, Nijhawan et al. achieved strong results in sentiment and emotion analysis using BERT,^41^ but their study did not extend to severity classification, nor did it provide interpretable outputs for end-users. Additionally, our study integrates explainability into a unified framework, combining multi-label emotion detection and multi-class severity prediction within a single platform. This sets our work apart from frameworks such as the FIRE-KNOP DES model, which, while achieving a detection accuracy of 88.33%, relied on smaller datasets and lacked practical deployment interfaces.^40^ Similarly, the Sentiment-Informed SBERT Ensemble achieved F1-scores of 76% on Reddit data^56^ but did not incorporate comprehensive explainability, further underscoring the distinctive value of our framework.

## Discussion

This study demonstrates that validation-weighted transformer ensembling consistently improves classification performance while maintaining transparency. The ensemble achieves top scores across various datasets, particularly excelling in minority or ambiguity-prone categories. The DepTformer-XAI-SV model provides (i) higher macro-F1 for minority labels, (ii) fewer adjacency errors between similar emotions, and (iii) reliable explanations that pass stability checks. For multi-label classification, it significantly improves the recognition of cognitive dysfunction and suicidal intent, with medium-to-large effect sizes and non-overlapping confidence intervals. These improvements stem from effective fusion and imbalance-aware training, not merely from lucky hyperparameter choices. Explanations are integral, with token-level attributions confirming that highlighted tokens genuinely support predictions, demonstrating stable behavior under resampling.

The gains are due to complementary inductive biases and reduced error covariance, not a single encoder’s strength. Different backbones exhibit varying capacities and biases, leading to lower error covariance, as shown by LOBO tests that highlight the utility of DeBERTa+BiLSTM. Slice analyses show that the ensemble effectively corrects common errors while aligning backbone influence with the imbalance-sensitive objective, outperforming uniform averaging. Quantitative analysis of explanations verifies their faithfulness and stability. Removing the top LIME-important tokens results in consistent drops in confidence, indicating a clear causal link between highlights and predictions. This is especially true for minority classes such as suicidal intent and cognitive dysfunction, which maintain moderate to high stability. Perturbation studies show that masking is the most reliable approach, while hard deletion offers slightly better AUC at the cost of increased variance.

This system is designed for decision support—not diagnosis—and to triage language that may indicate elevated risk. In practice, outputs should feed human-in-the-loop pathways: (i) surface high-risk cues to a trained reviewer, (ii) trigger stepped-care actions, and (iii) document rationale via XAI artifacts for audit. Deployment must respect efficiency constraints: the full ensemble suits batch/clinic back-ends, while lighter backbones enable low-latency prescreening; fallback modes and rate limits are required for limited compute. Fairness and privacy safeguards include de-identification at ingestion, encrypted storage, minimal data retention with opt-out, and routine bias monitoring across demographics with threshold recalibration if disparities emerge.

Qualitative analyses of true positives, false negatives, and borderline cases reveal that true positives have strong, clear positive weights, while false negatives often show mixed or unclear attributions. Overall, the findings suggest that (i) the ensemble’s token rationales are generally faithful, (ii) the stability is adequate for auditing and triage, and (iii) the explanation process makes ambiguities visible rather than obscured. Residual errors often arise in areas where signals are sparse, ambiguous, or subtle. Confusion frequently arises between similar emotions, such as worthlessness and hopelessness, where the difference lies more in perspective than in language. Pragmatic indirection can further obscure meaning, leading to mixed attributions and less reliable outcomes in borderline cases. Short posts worsen this issue, as the 21–40 token range lacks redundancy, allowing a few ambiguous words to heavily influence decisions. Cognitive complaints tend to be more about language context than consistent terminology, with suicide-risk language varying from direct to subtly implied. Even with ensemble methods, these categories show higher variability in accuracy and stability. Errors are widespread across the mild and moderate severity levels, highlighting challenges with subjective reporting.

Idiolect variations also create domain-shift issues; while XLM-RoBERTa helps in slang contexts, residual drift persists for niche groups. Three key design choices enhanced sensitivity to minority groups and stability. First, validation-weighted fusion based on macro-F1 outperformed uniform averaging, making model influence more sensitive to imbalances. Second, label-aware training improved recall for minority groups and sharpened decision boundaries, especially for rare emotions, where single encoders often overfit to majority trends. Third, controlled oversampling offered the best balance of precision and recall, as moderate duplication strengthened rare signal representations without losing precision. Generalization occurs along three dimensions. Transitioning from multi-label DepressionEmo to multi-class DSL resulted in a slight drop in micro-F1 but an increase in macro-F1 for the ensemble, indicating effective signal capture across emotional and clinical aspects.

Efficiency profiles support this operational transfer, while the ensemble can serve as a more accurate auditor or batch processor. The ensemble delivers the most robust minority-class performance and explanation stability, but at the cost of higher FLOPs, VRAM, and energy per 1k inferences. However, shifts to new platforms or cultures may hinder minority performance due to localized language use. Cross-lingual pretraining with XLM-RoBERTa aids this, but practical transfer benefits from lightweight adaptation. We prioritize data minimization and de-identification: no raw identifiers, masking of URLs and handles, and strict controls to prevent data leakage during training, validation, and testing. Even with de-identified social media data, there is a risk of re-identification through unique phrases, so outputs should avoid saving verbatim text and use hashed references instead. Bias may stem from demographic imbalances, cultural differences in expressing distress, and noise in minority labels.

The implications of our mental health assessment application are significant. It serves as a clinical decision-support tool that identifies linguistic cues related to depressive emotions and severity, providing clinicians with quick insights into a patient’s mental state. The system can be integrated into pre-consultation intake forms, allowing patients to describe their feelings, while clinicians receive a summary of emotional indicators before the session. It can also enhance tele-mental-health platforms by flagging high-risk language in messages between clients and therapists or helping triage teams prioritize messages with signs of suicidal intent. In stepped-care models, it aids in monitoring patients over time by analyzing periodic submissions to track emotional changes. The system provides clear, token-level explanations, enabling practitioners to contextualize results and avoid overreliance on automated decisions. However, ethical considerations are crucial, including protecting user privacy, ensuring de-identified interactions, preventing misuse for diagnostics, and maintaining human oversight. Deployment must comply with platform Terms of Service and clinical governance protocols, with clear data processing communication to preserve transparency and trust. With a modular architecture, the application also allows for future integration with diverse data sources and explainability techniques.

### Limitations of the study

This study has several key limitations. First, both datasets show significant class imbalance, especially in categories such as suicidal intent and mild depression. Despite using class-weighted losses and validation-weighted voting, performance on these minority labels was still low. This highlights biases in the training data and indicates a need for improved data interventions, such as advanced augmentation or synthetic sample generation using large language models. Second, the linguistic representation of symptoms such as cognitive dysfunction and hopelessness lacks consistent markers, complicating modeling efforts. While our approach captures contextual relationships well, text-only inputs miss important cues typically conveyed through voice tone, pauses, or facial expressions, leaving emotional signals partially undetected. Another limitation is related to explainability. Although LIME provides token-level attributions, its method has drawbacks. Explanations can vary across runs, often lack linguistic coherence, and may not accurately reflect the model’s reasoning, especially for more extended sequences or nonlinear dependencies common in transformer architectures. As a result, clinicians should view these outputs as supportive rather than definitive. Additional techniques, such as Integrated Gradients or SHAP, could help address some of these issues.

From a computational standpoint, large models such as DeBERTa and XLM-RoBERTa are resource-heavy, and ensembling increases both VRAM usage and inference time, making them harder to deploy in low-resource clinical settings or real-time applications. Additionally, the model’s generalizability is limited because it primarily trains on English-language, Western-centric social media texts. This may affect performance in multilingual or region-specific contexts, and challenges remain with slang, sarcasm, and informal dialects, despite XLM-RoBERTa’s cross-lingual training. Future work should focus on feasible and impactful improvements. Strategies such as model compression (e.g., quantization, pruning, and low-rank factorization) can reduce inference time and memory usage while preserving accuracy. Knowledge distillation could help create a lightweight model for mobile deployment. Enhancing generalizability may involve fine-tuning on regional data, unsupervised pretraining on in-domain texts, and culturally calibrating labels. To provide more precise explanations, future research should combine LIME with gradient- or attention-based interpretability methods and develop measures of stability. Ultimately, incorporating multimodal signals such as audio and facial cues is a longer-term objective, though initial efforts can focus on improving text-based approaches through continual learning and adaptive thresholding.

## Resource availability

### Lead contact

Information and requests for resources should be directed to and will be fulfilled by the Lead Contact, Abhishek Appaji (am.appaji@maastrichtuniversity.nl).

### Materials availability

The study did not generate new unique reagents.

### Data and code availability

• Data: The initial datasets used in this study are sourced from DepressionEmo,^27^ depression severity level (DSL),^57^ and depression signs detection (DSD).^58^ These datasets are publicly available from their original maintainers under research licenses.
• Code: All code and reproducibility scripts are publicly available at https://doi.org/10.5281/zenodo.18014776.
• Additional Information: Any additional information required to reanalyze the data reported in this article is available from the lead contact upon request.

## Acknowledgments

The authors gratefully acknowledge the support of their respective institutions and colleagues whose guidance and resources made this research possible. We also extend our thanks to the creators of the publicly available datasets used in this study, as well as to the broader research community for their valuable tools and contributions that informed and enabled this work.

This research did not receive any specific grant from funding agencies in the public, commercial, or not-for-profit sectors.

## Author contributions

**Sazzadul Islam**: writing – review and editing, writing – original draft, software, methodology, and conceptualization. **Rezaul Haque**: writing – review and editing, writing – original draft, software, visualization, methodology, and formal analysis. **Mahbub Alam Khan**: writing – review and editing, writing – original draft, software, visualization, methodology, and formal analysis. **Arafath Bin Mohiuddin**: writing – review and editing, writing – original draft, methodology, and data curation. **Md Ismail Hossain Siddiqui**: writing – review and editing, writing – original draft, visualization, and validation. **Zishad Hossain Limon**: writing – review and editing, writing – original draft, visualization, investigation, and conceptualization. **Katura Gania Khushbu**: writing – review and editing, writing – original draft, visualization, investigation, and conceptualization. **S M Masfequier Rahman Swapno**: writing - review and editing, investigation, formal analysis, validation, visualization, and conceptualization. **Md. Redwan Ahmed**: writing – review and editing, visualization, data curation, formal analysis, and conceptualization. **Abhishek Appaji**: writing - review and editing, investigation, formal analysis, validation, and supervision. All authors have read and approved the final article.

## Declaration of interests

The authors declare no competing interests.

## STAR★Methods

### Key resources table

**Table:** 

REAGENT or RESOURCE	SOURCE	IDENTIFIER
**Deposited data**		

DepressionEmo	Public repository	URL: https://github.com/abuBakarSiddiqurRahman/DepressionEmo/tree/main/Dataset
Depression severity level	Public repository	URL: https://github.com/usmaann/Depression_Severity_Dataset/blob/main/Reddit_depression_dataset.csv
Depression signs detection	Public repository	URL: https://github.com/rafalposwiata/depression-detection-lt-edi-2022/tree/main/data/original_dataset

**Software and algorithms**		

Code and web application	This study	GitHub: https://doi.org/10.5281/zenodo.18014776
DepTformer-XAI-SV	This study	Hyperparameters and training details reported in table, baseline models section
Python version 3.8	Python Software Foundation (PyPI)	https://www.python.org/downloads/release/python-3810/
PyTorch version 2.0.0	PyPI	https://pypi.org/project/torch/
TorchVision version 0.15.0	PyPI	https://pypi.org/project/torchvision/0.15.0/
Transformers version 4.36.0	PyPI	https://pypi.org/project/transformers/4.36.0/
Tokenizers version 0.14.1	PyPI	https://pypi.org/project/tokenizers/0.14.1/
Sentencepiece version 0.2.1	PyPI	https://pypi.org/project/sentencepiece/
Scikit-learn version 1.3.2	PyPI	https://pypi.org/project/scikit-learn/1.3.2/
Scipy version 1.10.1	PyPI	https://pypi.org/project/scipy/1.10.1/
Numpy version 1.23.5	PyPI	https://pypi.org/project/numpy/1.23.5/
Pandas version 2.1.1	PyPI	https://pypi.org/project/pandas/2.1.1/
Matplotlib version 3.7.2	PyPI	https://pypi.org/project/matplotlib/3.7.2/
Seaborn version 0.12.2	PyPI	https://pypi.org/project/seaborn/
Nltk version 3.8.1	PyPI	http://pypi.org/project/nltk/
Spacy version 3.7.2	PyPI	https://pypi.org/project/spacy/3.7.2/
Gensim version 4.3.2	PyPI	https://pypi.org/project/gensim/
Fasttext version 0.9.2	PyPI	https://pypi.org/project/fasttex/
Langid version 1.1.6	PyPI	https://pypi.org/project/langid/
Lime version 0.2.0.1	PyPI	http://pypi.org/project/lime/
Flask version 2.3.3	PyPI	https://pypi.org/project/Flask/
Torchserve version 0.9.0	PyPI	https://pypi.org/project/torchserve/
Torch-model-archiver version 0.9.0	PyPI	https://pypi.org/project/torch-model-archiver/
Requests version 2.31.0	PyPI	https://pypi.org/project/requests/
Regex version 2023.10.3	PyPI	https://pypi.org/project/regex/
Python-dotenv version 1.0.0	PyPI	https://pypi.org/project/python-dotenv/

**Other**		

GPU hardware for training/evaluation	NVIDIA RTX 3090	Reported in Table 7
Inference/efficiency benchmarks	This study	See Table 7

### Experimental model and study participant details

This study examines publicly available social media text to identify depression-related emotions and severity levels. No human participants were involved, and no personally identifiable information was accessed. All datasets were used in accordance with their original licenses and Terms of Service. There were no interventions or manipulations involving individuals. All model training and evaluation were conducted using these de-identified text datasets, in accordance with ethical standards for research with publicly available data.

### Method details

Our approach combines four diverse transformer backbones through a validation-based macro F1-weighted soft voting method, as shown in Figure 1. This fusion aligns with imbalance-sensitive objectives. To address class imbalance, we employ class-weighted loss functions, iterative stratified k-fold splits, and per-class threshold calibration for the multi-label task. We ensure full transparency in our training and hyperparameter search processes. Additionally, we compare our method against classical ML baselines, built on TF-IDF with optional averaged embeddings. For interpretability, we utilize LIME to provide token-level post-hoc attributions and validate the model’s faithfulness through token-deletion analyses. Moreover, we report both macro and micro metrics and conduct paired tests across folds with 95% confidence intervals. We also calculate effect sizes.

Let $D={\left\{\left(x_{i},y_{i}\right)\right\}}_{i=1}^{N}$ be a corpus of public, de-identified social-media posts, where $x_{i}$ is a tokenized post. We address two text classification tasks: For emotion detection, the label set $C_{e}=\left\{1,\ldots ,C_{e}\right\}$ contains $C_{e}=8$ depression-related emotions. Ground truth is a binary vector $y_{i}^{\left(e\right)}\in {\left\{0,1\right\}}^{C_{e}}$ (multiple emotions may co-occur). On the other hand, the severity assessment label set $C_{s}=\left\{1,\ldots ,C_{s}\right\}$ contains $C_{s}=4$ mutually exclusive severity levels, with ground truth $y_{i}^{\left(s\right)}\in C_{s}$. For each backbone model m∈{1,…,M}, let $z_{m}\left(x\right)$ denote class logits and $p_{m}\left(x\right)$ the corresponding probabilities. The ensemble probability $\hat{p}\left(x\right)$ is used for decisions below. For the multi-label task (Equation 1), predictions are obtained with per-class thresholds $\tau_{c}$, where $\left\{\tau_{c}\right\}$ are calibrated on the validation split to maximize macro-F1 per class. For the multi-class task, we predict the most probable class using Equation 2. To reduce bias toward frequent classes, we use class weights $w_{c}$ computed on each training fold (e.g., inverse class frequency, normalized). These weights enter the losses for both tasks. For the multi-label task, we minimize weighted binary cross-entropy (Equation 3), where σ(·) is the sigmoid. For the multi-class task, we minimize weighted cross-entropy using Equation 4.

(Equation 1) $${\hat{y}}_{i,c}^{\left(e\right)}=1\left[{\hat{p}}_{i,c}^{\left(e\right)}\geq \tau_{c}\right],c\in C_{e}\text{,}$$

(Equation 2) $${\hat{y}}_{i}^{\left(s\right)}=\arg\;\max_{c\in C_{s}}{\hat{\;p}}_{i,c}^{\left(s\right)}\text{.}$$

(Equation 3) $$L_{\text{ML}}=-\frac{1}{C_{e}}\sum_{c=1}^{C_{e}}w_{c}\left[y_{c}\;\log\;\sigma \left(z_{c}\right)+\left(1-y_{c}\right)\log\left(1-\sigma \left(z_{c}\right)\right)\right]\text{,}$$

(Equation 4) $$L_{\text{MC}}=-\sum_{c=1}^{C_{s}}w_{c}\;1\left[y=c\right]\;\log\;\text{softmax}{\left(z\right)}_{c}\text{.}$$

We use k-fold cross-validation with stratification; for the multi-label task, folds are constructed via iterative stratification so that label co-occurrences are preserved. Thresholds $\left(\tau_{c}\right)$ are selected on each validation fold and then fixed for test evaluation within that fold. Because both tasks are imbalanced and minority classes are clinically salient, we prioritize macro-F1 for model selection and reporting, and we also provide micro-F1 and per-class precision/recall/F1 in Results.

#### Datasets

We study social-media text for two complementary tasks: (i) multi-label emotion detection in depression-related posts using the DepressionEmo dataset; and (ii) single-label severity classification (Minimal/Mild/Moderate/Severe) using a Merged Depression Severity Detection (MDSD) dataset that combines two clinical-anchored corpora. Both datasets are publicly available under their original licenses; no attempt was made to re-identify users.

DepressionEmo dataset consists of 6,037 Reddit posts that represent a wide range of emotions associated with depression. Labels were produced via a two-stage process: automatic pre-labeling with a pretrained model followed by expert review and adjudication. All posts were de-identified and handle/user metadata removed. Each post is categorized into one of eight emotional states associated with depression: anger, cognitive dysfunction (forgetfulness), emptiness, hopelessness, loneliness, sadness, suicidal intent, and worthlessness. These categories have been chosen to reflect the slight emotional experiences of individuals dealing with depression. Figure 2A shows the class distribution of the dataset. It captures the complexity of emotions related to depression, with sadness accounting for the largest proportion at 21.2%, followed by hopelessness at 19.1%. Other well-represented emotions include loneliness (12.7%), worthlessness (13.6%), anger (11.1%), and emptiness (10.3%). Less common emotions, such as cognitive dysfunction (5.2%) and suicidal intent (6.8%), provide critical insights into the more severe manifestations of depression. The emotional diversity of this dataset enhances its usefulness for analyzing linguistic patterns and understanding how individuals express their struggles online. From an ethical standpoint, the DepressionEmo dataset adheres to strict privacy guidelines. All user information has been anonymized to prevent identification.

Below the table displays the statistical analysis of the dataset, highlighting a significant class imbalance. The Sadness category has the highest number of posts, totaling 4,665, while the cognitive dysfunction category has the fewest, with only 1,148 posts. The text length is generally consistent across categories, with average lengths ranging from 506.54 characters for Worthlessness to 528.28 characters for Suicide Intent. However, categories such as Sadness and Suicide Intent also include some of the longest maximum text lengths, reaching up to 1,910 characters. Vocabulary size varies among categories; Sadness has 16,321 unique words, and Suicide Intent has 17,194, indicating greater linguistic diversity. In contrast, cognitive dysfunction has the smallest vocabulary, with only 8,008 unique words. Word counts are fairly uniform across categories, averaging between 116.87 words for Hopelessness and 121.86 words for Emptiness, demonstrating consistency in tokenization. Furthermore, the stopword ratio remains stable across all categories, ranging from 0.461 to 0.470.

**Table:** Statistical analysis of the DepressionEmo dataset

Statistics	Cognitive Dysfunction	Emptiness	Hopelessness	Loneliness	Sadness	Suicide Intent	Worthlessness
Total label assignments	1148	2272	1844	2802	4665	4546	2991
Average text length	508.05	523.7	521.24	524.28	527.88	528.28	506.54
Minimum text length	26	20	30	20	20	20	26
Maximum text length	1910	1137	1493	1146	1910	1910	1910
Median text length	492	512	508	513	510	514	489
Avg Word Count	117.65	121.86	116.87	121.44	121.65	120.16	117.53
Vocabulary Size	8008	10418	11494	12138	16321	17194	12498
Stopword Ratio	0.463	0.467	0.461	0.467	0.470	0.467	0.468

Figure 3 shows a Pearson correlation heatmap that illustrates the interrelationships among eight multi-label emotional categories within the dataset. Strong positive correlations are evident among emotional states that reflect depressive internal experiences, specifically “Worthlessness,” “Hopelessness,” “Emptiness,” and “Loneliness.” For instance, the correlation between “Worthlessness” and “Hopelessness” is 0.49, indicating considerable linguistic overlap, suggesting that respondents often express these emotions at the same time. Similarly, “Loneliness” and “Emptiness” also have a strong correlation of 0.49, reflecting common themes of isolation and emotional voids that frequently appear together in narratives of depression. On the other hand, “Anger” and “Cognitive Dysfunction” show lower values with other emotions, indicating that they possess unique linguistic expressions and contexts within the dataset. The minimal correlation of “Anger” with “Sadness” (0.07) and its slightly negative correlation with “Loneliness” (−0.05) present different emotional dynamics. This difference likely arises because anger tends to be externalized and expressed through frustration, in contrast to the internally directed emotional states like Loneliness or Hopelessness.

Interestingly, “Suicide Intent” exhibits only weak correlations with “Sadness” (0.15) and “Hopelessness” (0.24). Expressions of suicidal ideation have specific linguistic markers that indicate a severe state, featuring clear or subtle references that differ from general descriptions of depression. Recognizing these subtle language differences is important for accurate modeling, as overlooking them can impair the detection of high-risk emotional indicators. These findings focus the need to differentiate closely related emotional states from uniquely expressed emotions in models. By incorporating nuanced language patterns, we can improve the sensitivity and specificity of models, particularly in identifying severe emotional distress, such as suicidal intent.

We combined two publicly available English-language datasets dedicated to the detection of the severity of depression: Depression severity level (DSL)^57^ and depression signs detection (DSD).^58^ Both are publicly available; links and retrieval instructions are provided in data and code availability section. All content is de-identified and used for research (non-diagnostic) in compliance with licenses and Terms of Service. The DSL dataset provides quantitative assessments of depression severity using two established metrics: the Depression Severity Annotation Schema (DSAS) and the Beck Depression Inventory (BDI-3). BDI-3 is a widely used self-report tool that evaluates the degree to which symptoms of depression— such as mood, behavior and physical health—are felt by the respondents; scores range between 0 and 63. The DSAS further categorizes these scores into four severity levels: 0–9, 10–18, 19–29, and 30–63. We harmonize both corpora to the shared four-level scheme, deduplicate overlaps via URL and near-duplicate hashing (MinHash, Jaccard ≥0.9), filter to English (langid ≥0.95), and retain one label per post (single-label task). Conflicting labels are adjudicated.

The final merged MDSD contains 13,804 posts: Minimal (6,090 instances), Mild (290 posts), Moderate (6,174 posts), and Severe (1,250 posts). Figure 2B shows the per-class distribution and class imbalance. As severity increases, posts tend to be longer and lexically richer (below table). The Moderate category has the highest number of posts, while the Mild severity category is significantly underrepresented, with only 290 posts. As severity increases, both the average text length and word count also increase. Severe posts display the highest averages, with 1,018.25 characters and 228.78 words, indicating more detailed narratives compared to the shorter posts found in the Mild category, which average 477.75 characters and 105.2 words. In terms of vocabulary size, the Moderate severity category has the largest at 24,039 words, while the Mild severity category has the smallest vocabulary size at 3,768 words. This indicates a richer linguistic diversity in the Moderate category and a more limited lexical variety in the Mild category. Despite these differences in content, the ratio of stopwords remains consistent across all categories, ranging from 0.471 to 0.49.

**Table:** Statistical analysis of the MDSD dataset

Statistics	Minimal	Moderate	Mild	Severe
Total posts	7236	10888	290	1771
Average text length	517.46	841.95	477.75	1018.25
Minimum text length	6	15	154	16
Maximum text length	19684	27432	1639	15996
Median text length	370	545	450	647
Avg Word Count	115.85	190.63	105.2	228.78
Vocabulary Size	22426	24039	3768	12725
Stopword Ratio	0.471	0.477	0.49	0.471

#### Data preprocessing

Social media data is often unstructured and noisy, containing misspellings, informal language, and irrelevant content that can hinder natural language processing (NLP) models. Preprocessing the data is essential for ensuring it is clean and meaningful, allowing the models to focus on important patterns.^59^^,^^60^ By applying various text preprocessing techniques (Figure 4), we transformed the noisy social media text into a structured format. Each step addresses specific challenges, such as ensuring consistency, removing irrelevant elements, and highlighting linguistic patterns. An 80–5–15 split was used to divide both datasets into training, validation and testing subsets.

We applied a transformer-friendly pipeline designed to preserve semantic cues while standardizing obvious noise. The same steps were used for both datasets. We lowercased all text to reduce sparsity from capitalization while retaining proper nouns via subword units. Tokenization was performed by the corresponding transformer tokenizer (e.g., WordPiece/BPE), which natively handled punctuation and contractions; therefore, we did not strip punctuation prior to tokenization to avoid degrading lexical context. To normalize inflectional variants without harming semantics, we applied lemmatization (via POS-aware rules) to the plain-text copy used for auxiliary statistics and classic ML baselines. For transformer inputs, we avoided lemmatizing the final sequence passed to the model; instead, we relied on the pretrained tokenizer. We avoided stemming because it could over-truncate subwords (e.g., depress” from depression”/“depressive”), which conflicted with subword tokenization and could distort class-indicative morphemes. We normalized platform artifacts rather than removing them: all http(s)://… links → <URL>, user mentions (e.g., u/handle, @handle) → <USER>, and numbers → <NUM>. This preserved the presence of these cues while preventing lexical sparsity and limiting inadvertent leakage of identifiers.

Emojis and common emoticons were retained because they carried sentiment signal. When unsupported by the tokenizer, they were mapped to descriptive placeholders (e.g., <SMILE>, <SAD>) rather than dropped. To reduce noise from extremely short or empty posts, we discarded texts with fewer than 20 non-whitespace characters or three tokens (computed post-tokenization). These values were chosen on the validation set to balance coverage and quality. We used a standard English stop-list to reduce dimensionality; this difference was noted in the Baselines section for reproducibility. We did not prune rare tokens for transformer inputs (handled upstream by subword vocabularies). For classical baselines, we pruned terms with corpus frequency <, 2 and applied conservative spell correction for common typos, avoiding changes to domain-specific terms (e.g., medication names). HTML tags were stripped, and whitespace was normalized. Near-duplicate posts were removed before cross-validation to prevent leakage.

We address label imbalance through (i) class-weighted losses, (ii) stratified cross-validation (iterative for multi-label), (iii) training-fold oversampling, and (iv) per-class threshold calibration for multi-label predictions. Weights $w_{c}$ are computed on each training fold (inverse-frequency, normalized) and applied to the multi-label and multi-class cross-entropy losses. We use k-fold cross-validation with identical folds across systems. For emotions we adopt iterative stratification to preserve class frequencies and label co-occurrences; for severity we use standard stratification. We perform random oversampling of minority classes on the training fold with a cap ratio r (e.g., minority up to r=0.5 of the majority), leaving validation/test untouched to avoid leakage. Regularization (dropout, weight decay) and early stopping limit overfitting. For each class c, we select $\tau_{c}$ on the validation split by maximizing class-wise F1 over a grid $\tau_{c}\in \left[0.3,0.7\right]$; the calibrated thresholds are then fixed for that fold’s test partition.

#### Feature extraction

This study trains models with a hybrid embedding approach. Instead of relying on a neural network to continuously learn available inputs, GloVe generates fixed-size vectors by modeling statistical co-occurrence of words within a large corpus. In essence, the thought is that the ratio of word cooccurrence probabilities retains semantic information. Given a pair of words $w_{i}$ and $w_{j}$, GloVe optimizes the following cost function (Equation 1). Where, $X_{ij}$ is the co-occurrence count of words and $f\left(X_{ij}\right)$ is a weighting function to handle the influence of frequent and rare words. Furthermore, $b_{i}$ and $b_{j}$ are bias terms and V is the vocabulary size. GloVe takes advantage of the global semantic relationship among words, which makes it appropriate for handling contextual and semantic search over textual data.^61^

(Equation 5) $$J=\sum_{i,j=1}^{V}f\left(X_{ij}\right){\left(w_{i}^{⊤}w_{j}+b_{i}+b_{j}-\log\left(X_{ij}\right)\right)}^{2}$$

FastText generates word embeddings by considering subword information through character n-grams. Each word (w) is represented as a bag of its subword n-grams, enabling the model to construct embeddings for OOV words based on their subword composition. For a word, the embedding is calculated using Equation 2. Where, N(w) is the set of n-grams for word (w) and (g) is the vector representation of each n-gram. FastText’s subword-based approach allows it to handle rare and morphologically rich words, which is especially valuable for noisy, informal social media data.^62^

(Equation 6) $$w=\frac{1}{\left|N\left(w\right)\right|}\sum_{g\in N\left(w\right)}g$$

The hybrid embedding approach integrates the global semantic relationships captured by GloVe with the subword-level robustness of FastText. Pre-trained embeddings for GloVe and FastText were obtained from publicly available corpora, such as Common Crawl and Wikipedia. To produce consistency, the vocabularies of GloVe and FastText were aligned according to Equation 2. Then, we got vectors of a same word if it exists in both vocabularies. One vocabulary dynamically generated its vector using subword n-grams if word was missing. The hybrid embedding vector was constructed by taking the concatenation of both the vectors, for each word.

(Equation 7) $$v_{\text{hybrid}}\left(W\right)=v_{\text{GloVe}}\left(W\right)⊕v_{\text{FastText}}\left(W\right)$$

We concatenate GloVe (300days) and FastText (300days) into a 600-dim vector; OOV terms fall back to FastText subword; vectors are L2-normalized before concatenation. There are also several advantages of the hybrid embedding approach, for which it can work better to the depression detection task. As a first step, GloVe exploits global patterns of word co-occurrence and thus encodes a powerful semantic understanding of the text. This is necessary to find overall sentiment and contextual relationship in depression-related language. Second, FastText uses the subword information to complement GloVe. The combination of these two methods results in better methods to represent text through hybrid embeddings. The model integrates global context with subword-level details of depression, thus helping it to pick up on fine nuances of speech that are indicative of depression-related emotional and linguistic patterns. Moreover, the hybrid approach benefits from FastText’s robustness to informal language, which makes the approach optimal for noisy data found in social media.

#### Baseline models

To quantify the incremental value of transformer ensembling, we include strong classical text classifiers trained on transparent features. These baselines establish performance anchors under limited capacity and provide interpretability via feature weights. We evaluate (i) Logistic Regression (LR) with $l_{2}$ regularization (multinomial for multi-class, one-vs-rest for multi-label), (ii) Linear SVM (hinge loss, one-vs-rest), and (iii) Random Forest (RF) with class-balanced sampling. For multi-label emotion detection, LR/SVM adopt a one-vs-rest scheme with independent decision functions per label; for severity (multi-class), all models predict a single class. Our primary representation is TF–IDF with 1–2 g, sublinear TF scaling, English stopword removal, and min−df=2. Vectors are $l_{2}$-normalized. As an auxiliary representation, we report averaged word embeddings of dimension d: each post vector is the mean of in-vocabulary token embeddings; out-of-vocabulary tokens map to ⟨UNK⟩. When used, embedding features are z-normalized per dimension. Exact dimensions and pretrained sources are listed in below table.

**Table:** Hyperparameter search spaces and selection criteria for classical ML and RNN baselines

Model/Component	Hyperparameters
**ML baselines**	

LR	*C* ∈ {10^−3^, 10^−2^, 10^−1^, 1, 10}; penalty = $l_{2}$; solver = liblinear/lbfgs
Linear SVM	*C* ∈ {10^−3^, 10^−2^, 10^−1^, 1, 10}; loss = hinge/sq.-hinge; class_weight = balanced
RF	trees ∈ {200, 500, 1000}; max_depth ∈ {*None*, 20, 50}; max_features = {sqrt,log2}
TF–IDF features	n-grams = {1,2}; min-df = {1,2,5}; *χ*^2^-*k* = {10^4^, 5×10^4^, all}
Averaged embeddings	source = {GloVe, FastText}; dim = *d* ∈ {300, 600}; pooling = mean; OOV = ⟨UNK⟩

**Deep learning baselines (RNN family)**	

LSTM	layers ∈ {1, 2}; units per layer ∈ {128, 256}; activation = {tanh, relu}
BiLSTM	bidirectional = true; units ∈ {(256, 128), (256, 256)}; activation = {tanh, relu}
GRU	layers ∈ {1, 2}; units per layer ∈ {128, 256}; activation = {tanh, relu}
BiGRU	bidirectional = true; units ∈ {(256, 64), (256, 128)}; activation = {tanh, relu} Embedding layer hybrid (GloVe+FastText); dim = *d*_ℎ_ ∈ {300, 600}; trainable = {true, false}; OOV = ⟨UNK⟩ Dropout embedding/feature dropout ∈ {0.2, 0.3, 0.5}; recurrent dropout ∈ {0, 0.2}
Output heads	multi-label: sigmoid; multi-class: softmax; class weights = balanced Thresholding multi-label per-class *τ*_*c*_ ∈ [0.3, 0.7] (grid); select by class-wise F1 on validation

Best settings are chosen on the inner validation of each training fold to maximize macro-F1.

For LR and SVM we use class_weight = ‘balanced’, which scales per-class loss by the inverse of class frequency in the training fold. For RF, we use class-balanced sampling or class weights. For multi-label LR/SVM, class weights are set independently for each one-vs-rest classifier. For LR, we use predicted probabilities; for SVM, we apply Platt scaling on the validation split to obtain calibrated probabilities. Per-class decision thresholds $\tau_{c}$ are selected on the validation fold to maximize macro-F1. We perform inner validation on the training fold to select hyperparameters, then report results under the outer k-fold protocol with identical folds as neural models for fairness. Selected settings maximize validation macro-F1 (ties broken by micro-F1) and are then fixed within each outer fold.

We stored the hybrid embeddings of all vocabulary words in an embedding matrix, which served as the input layer for our deep learning models. Each row of this matrix represents a word’s hybrid vector. To establish baseline models, we experimentally evaluated several architectures of RNNs. The Long Short-Term Memory (LSTM) network, which features three gates (forget, input, and output) regulates the flow of data and preserves relevant information over time steps. Our experiments indicated that using two LSTM layers, each with 256 neurons and employing the ReLU activation function, resulted in the best capture of text features. This configuration efficiently processed non-negative inputs and effectively modeled non-linear relationships. The Bidirectional LSTM (Bi-LSTM) enhances the LSTM architecture by processing sequences in both directions, allowing it to capture both past and future contexts for improved long-range dependency modeling. We implemented two Bi-LSTM layers with 256 and 128 units, respectively. We found that reducing the number of units in the second layer improved classification accuracy. Similar to LSTM, Bi-LSTM effectively mitigates the vanishing gradient problem. The Gated Recurrent Unit (GRU) simplifies the LSTM architecture by utilizing only update and reset gates. This results in faster training times, although it has slightly less capacity for handling long-term dependencies. Our Bi-GRU model consisted of two layers with 256 and 64 units, respectively, and also used the ReLU activation function. The reduced number of units in the second layer helped minimize overfitting and improve efficiency. While GRUs sacrifice some memory capability for increased speed, they are well-suited for tasks that require rapid computation.

#### Backbone models and selection rationale

We employ four transformer backbones: DistilBERT, ALBERT, XLM-RoBERTa, and DeBERTa to capture complementary inductive biases in efficiency, parameter sharing, cross-lingual robustness, and local sequence modeling. This architectural and pretraining diversity is intended to reduce pairwise error correlation and improve macro-F1 on minority classes.

DeBERTa-BiLSTM model combines the contextual representation strength of DeBERTa (Disentangled Bidirectional Encoder Representations from Transformers) with the sequential modeling power of BiLSTM. As shown in Figure 5, the input text is tokenized into $X_{1},X_{2},\ldots ,X_{n}$, augmented with special tokens [CLS] and [SEP], and passed through DeBERTa’s embedding layer. This layer integrates token embeddings (E), positional embeddings (T), and disentangled attention mechanisms to produce rich contextual representations.^63^ These embeddings capture both syntactic and semantic relationships between words. The encoder output is averaged to form a compact contextual vector per token,^64^ which is then passed through a BiLSTM layer. The BiLSTM processes the sequence bidirectionally, producing hidden states $\left(h_{t}\right)$ that integrate forward $\left(h_{t}^{+}\right)$ and backward $\left(h_{t}^{-}\right)$ dependencies. The final hidden state is then fed into a fully connected layer that maps the sequence representation to n class logits $\left(y_{1},y_{2},\ldots ,y_{n}\right)$ for depression-level classification.

ALBERT introduces two core techniques—parameter sharing across layers and factorized embedding parameterization—that reduce memory usage while maintaining strong contextual understanding.^65^ As depicted in Figure 6, the model receives tokenized sequences that are converted into token and positional embeddings. These embeddings, which represent token identity and order, are processed through encoder layers optimized for efficiency. Unlike BERT, ALBERT decouples the embedding and hidden dimensions, enabling smaller embeddings with large hidden layers for reduced computation.^66^ The transformer layers employ multi-head self-attention to capture global dependencies across the sequence. Parameters are shared across layers, further reducing redundancy. The representation of the [CLS] token serves as the global sequence embedding, which passes through a feedforward layer to predict the depression severity class.

Xlm-ReBERTa extends RoBERTa to multilingual pretraining, enabling cross-lingual understanding for depression detection.^67^ Figure 7 shows its structure for processing premise–hypothesis input pairs. Each sequence is tokenized, segmented with special tokens, and enriched with positional embeddings before being processed by stacked transformer layers. The encoder employs multi-head self-attention to learn contextual dependencies and generate bidirectional embeddings $T_{1},T_{2},\ldots ,T_{n}$ containing semantic and syntactic cues. The [CLS] representation $\left(C_{\left[CLS\right]}\right)$ from the final layer is used as the sequence-level embedding. This is projected through a linear layer to produce class logits $C_{\text{NLI}},C_{P},C_{F}$, representing depression severity predictions.

DistilBERT is a compact, efficient variant of BERT trained via knowledge distillation, achieving about 97% of BERT’s accuracy with 40% fewer parameters.^68^ As illustrated in Figure 8, it tokenizes input text using the WordPiece tokenizer, converts tokens into embeddings (token and positional), and processes them through a reduced number of transformer layers (M<N). The model inherits contextual richness from BERT by learning from its output distributions, hidden states, and attention patterns. Multi-head self-attention captures long-range dependencies, while layer normalization stabilizes training. The reduced architecture maintains high-quality contextual embeddings, which are passed through a classification layer to produce logits corresponding to depression severity levels.

Our backbone set is intentionally heterogeneous along three axes: (i) parameterization and capacity (lightweight vs. larger encoders), (ii) pretraining data and objectives (general English vs. cross-lingual corpora; parameter-shared vs. standard), and (iii) architectural bias (pure transformer vs. transformer augmented with a BiLSTM head for local order sensitivity). Below table summarizes these contrasts. The goal is to decorrelate residual errors so that different models excel on different linguistic regimes typical of social-media mental-health text. Let $\hat{p}\left(x\right)=\sum_{m}\alpha_{m}p_{m}\left(x\right)$ be the ensemble probability with normalized weights $\alpha_{m}$. For any class score, the variance of the ensemble is formulated in Equation 8.

(Equation 8) $$\text{Var}\left[\sum_{m}\alpha_{m}p_{m}\right]=\sum_{m}\alpha_{m}^{2}\;\text{Var}\left[p_{m}\right]+2\sum_{i<j}\alpha_{i}\alpha_{j}\;\text{Cov}\left(p_{i},p_{j}\right)$$

**Table:** Backbone diversity and resource profile

Backbone	Pretraining/bias	Head	Params (M)	VRAM (GB)	Latency (ms/post)	Strength targeted
DistilBERT	Distilled BERT (general English)	Linear	66	3.2	22.4	Efficiency, fast inference
ALBERT	Factorized emb., cross-layer sharing	Linear	12	2.7	19.1	Parameter-efficiency, regularization
XLM-RoBERTa	Cross-lingual masked LM (Base)	Linear	270	5.1	34.8	Robustness to slang/code-mixing
DeBERTa	Disentangled attention; local order via BiLSTM	BiLSTM → Linear	142	4.6	39.7	Token-order sensitivity, subtle shifts

Reducing pairwise covariance through model diversity lowers the second term, improving stability and, empirically, macro-F1 on minority classes where single models are brittle. As presented in above table, DistilBERT/ALBERT contribute efficiency and regularization, XLM-RoBERTa adds robustness to lexical variation and code-mixing, and DeBERTa with a BiLSTM head provides stronger token-order sensitivity. The four models differ in three main areas: parameter count, pretraining data and objectives, and architectural design. These variations aim to reduce residual errors. For example, ALBERT’s parameter sharing helps prevent overfitting, XLM-RoBERTa is resilient to slang and spelling variations from social media, and DeBERTa with BiLSTM captures local order and sentiment shifts effectively. DistilBERT offers quick and stable predictions, enhancing confidence in ensemble outcomes. Altogether, this diversity is expected to improve macro-F1 scores, particularly for minority labels, by decreasing prediction covariance. DistilBERT and ALBERT excel in efficiency and can function as low-latency options, while XLM-RoBERTa and DeBERTa+BiLSTM provide robustness for tackling challenging classes. This ensemble approach combines these strengths without the burden of a single large model, making it suitable for production environments that need to identify rare but important categories while managing latency and memory constraints.

#### DepTformer-XAI-SV execution process (ensemble fusion)

The ensemble aggregates backbone probabilities via a weighted average whose weights are static and derived from each backbone’s validation performance. Concretely, each transformer $C_{i}$ produces a probability vector $P_{i}\left(x\right)=\left[p_{i1},\ldots ,p_{ic}\right]$ over c classes with $\sum_{j=1}^{c}p_{ij}=1$. We compute a scalar score $s_{i}$ on the validation split (macro-F1 unless stated otherwise) and set the unnormalized weight ${\tilde{W}}_{i}=s_{i}$; normalized weights are $W_{i}={\tilde{W}}_{i}/\sum_{k}{\tilde{W}}_{k}$. The fused probability for class j is then Equation 9. For the multi-class task, the prediction is ${\mathrm{arg max}}_{j}\;P_{j}\left(x\right)$. For the multi-label task, we apply per-class thresholds, ${\hat{y}}_{j}=1\left[P_{j}\left(x\right)\geq \tau_{j}\right]$, where $\tau_{j}$ are selected on the validation split to maximize class-wise F1 (grid over [0.3,0.7]). This validation-weighted soft vote is simple and stable, aligns fusion with our imbalance-sensitive objective (macro-F1), and avoids overfitting risks of training an additional meta-learner, while retaining the interpretability of probability averaging.^69^ As illustrated in Figure 9, the weights are estimated once per fold from validation performance and remain fixed for test-time aggregation.

Let the ensemble consist of n classifiers $C_{1},\ldots ,C_{n}$, each outputting $P_{i}=\left[p_{i1},\ldots ,p_{ic}\right]$ with Equation 10. We define the normalized static weights as Equation 11 and aggregated class probabilities and decisions following Equations 12 and 13. In practice we compute $W_{i}$ on the validation split of each outer fold and keep them fixed for that fold’s test partition to preserve strict train/validation separation. In the next section, we reported an ablation of uniform versus validation-weighted fusion and alternative weighting criteria. The complete methodology is outlined in Algorithm 1 (see Data S1), which details preprocessing, model training, validation scoring for weight computation, threshold calibration (multi-label), and final prediction aggregation.

(Equation 9) $$P_{j}\left(x\right)=\sum_{i=1}^{n}W_{i}\;p_{ij}\left(x\right)$$

(Equation 10) $$\sum_{j=1}^{c}p_{ij}=1,\forall i\in \left\{1,\ldots ,n\right\}\text{.}$$

(Equation 11) $$W_{i}=\frac{F1_{\text{val}}^{\text{macro}}\left(C_{i}\right)}{\sum_{k=1}^{n}F1_{\text{val}}^{\text{macro}}\left(C_{k}\right)}\text{.}$$

(Equation 12) $$P_{j}\left(x\right)=\sum_{i=1}^{n}W_{i}p_{ij}\left(x\right),{\hat{y}}_{\text{multi}\text{-}\text{class}}=\arg\;\max_{j\in \left\{1,\ldots ,c\right\}}P_{j}\left(x\right)\text{,}$$

(Equation 13) $${\hat{y}}_{\text{multi}-\text{label},j}=1\left[P_{j}\left(x\right)\geq \tau_{j}\right],\;j\in \left\{1,\ldots ,c\right\}\text{.}$$

This mechanism ensures that higher-performing classifiers exert greater influence while weaker ones are down-weighted, reducing variance and improving stability on minority labels. The selection of diverse transformers further supplies complementary inductive biases—contextual, syntactic, and sequential—which the ensemble exploits to enhance robustness.^70^ DistilBERT provides lightweight, responsive predictions^71^; ALBERT contributes parameter efficiency and regularization^72^; XLM–RoBERTa adds robustness to varied, informal social-media language^73^; and DeBERTa–BiLSTM strengthens local order modeling.^63^ Together, validation-weighted soft voting integrates these strengths into a single, reliable predictor.

#### Training configuration

All experiments were conducted on a system with an NVIDIA RTX 3090 (24 GB VRAM), 128 GB RAM, and an AMD Ryzen 9 5950X CPU running Ubuntu 20.04 LTS. Models were implemented in Python 3.9 using PyTorch 2.0 and HuggingFace Transformers 4.36. Mixed precision was used to reduce memory and improve throughput. The web application was developed with Flask and TorchServe on CUDA 11.8. For inference-only reproduction, a GPU with at least 8 GB VRAM and 16 GB RAM is sufficient; full training is most reliable with 16 GB VRAM and SSD storage.

We adopt a common training recipe across backbones and tasks to ensure fair comparison. Optimizer is AdamW with linear-decay learning-rate scheduling and warm-up. Core settings include initial learning rate, batch size, maximum epochs, dropout, weight decay, gradient clipping, early stopping on validation macro-F1, fixed seeds, maximum sequence length, and optional mixed precision. Early stopping on macro-F1 aligns model selection with the imbalance-sensitive objective. We perform a bounded random search within predefined ranges (below table); the search budget is fixed per model and per task. For each outer fold, candidate settings are trained on the training split and selected on the validation split using macro-F1 as the criterion (ties broken by micro-F1 and then latency). The selected configuration is frozen and evaluated on that fold’s test split. This protocol is identical for all backbones and baselines.

**Table:** Combined training and hyperparameter search

Hyperparameter	Range	Emotion (selected)	Severity (selected)
**Training configuration**			

Optimizer	{Adam, AdamW}	AdamW	AdamW
Learning rate (initial)	{1e-5, 2e-5, 3e-5, 5e-5}	2e-5	2e-5
Schedule	{linear, cosine} warm-up {0,10,20%}	linear; 10% warm-up	linear; 10% warm-up
Batch size	{8, 16, 32}	16	16
Max epochs	{10, 20, 50}	10	10
Early stopping (patience)	{2, 3, 5} on val macro-F1	3	3
Dropout	{0.1, 0.3, 0.5}	0.3	0.3
Weight decay	{0, 0.01, 0.05}	0.01	0.01
Gradient clip	{no clip, 1.0}	1.0	1.0
Max sequence length	{128, 256, 384}	256	256

**Task-specific**			

Threshold grid (multi-label)	[0.3, 0.7], step 0.05	per-class *τ*_*c*_	–
Loss (multi-label)	fixed	weighted BCE	–
Loss (multi-class)	fixed	–	weighted cross-entropy
Search budget (trials/model)	fixed	12 random trials	12 random trials

Identical folds, preprocessing, and search protocol are used across all models. The multi-label task uses iterative stratification; the multi-class task uses standard stratification. No test information is used during search, calibration, or fusion. We fix random seeds per fold and per trial, and record all chosen hyperparameters and command lines. Where feasible, we enable deterministic cuDNN operations and document known nondeterminisms.

### Quantification and statistical analysis

#### Evaluation

Let C={1,…,C} denote the class set. For each class c∈C, let $TP_{c},FP_{c},FN_{c},TN_{c}$ be the contingency counts accumulated over all examples in a fold. Predictions for the multi-class task are obtained by argmax; for the multi-label task, predictions are thresholded per class using calibrated $\left\{\tau_{c}\right\}$. To avoid division by zero, for each class c we add a small ε>0 to denominators when necessary (Equation 14). Micro-averaged metrics are computed using Equation 15.

(Equation 14) $$P_{c}=\frac{TP_{c}}{TP_{c}+FP_{c}+\varepsilon },R_{c}=\frac{TP_{c}}{TP_{c}+FN_{c}+\varepsilon },F1_{c}=\frac{2P_{c}R_{c}}{P_{c}+R_{c}+\varepsilon }\text{.}$$

(Equation 15) $$P^{\text{macro}}=\frac{1}{C}\sum_{c=1}^{C}P_{c},R^{\text{macro}}=\frac{1}{C}\sum_{c=1}^{C}R_{c},F1^{\text{macro}}=\frac{1}{C}\sum_{c=1}^{C}F1_{c}\text{.}$$

Micro-averaged metrics were computed using Equation 16, where $TP_{\text{micro}}=\sum_{c}TP_{c}$, $FP_{\text{micro}}=\sum_{c}FP_{c}$, and $FN_{\text{micro}}=\sum_{c}FN_{c}$. All statistical comparisons are conducted on the same outer folds. For each pair of systems, we compute fold-wise differences on macro-F1 and apply a paired t-test when the differences are approximately normal. For each class, we also report 95% confidence intervals and effect sizes: Cohen’s d for approximately normal differences and Cliff’s Δ. Multiple comparisons are controlled via paired t-tests. Primary inferences use macro-F1; secondary analyses include micro-F1 and minority-class macro-F1 subsets.

(Equation 16) $$P^{\text{micro}}=\frac{TP_{\text{micro}}}{TP_{\text{micro}}+FP_{\text{micro}}+\varepsilon },R^{\text{micro}}=\frac{TP_{\text{micro}}}{TP_{\text{micro}}+FN_{\text{micro}}+\varepsilon },F1^{\text{micro}}=\frac{2P^{\text{micro}}R^{\text{micro}}}{P^{\text{micro}}+R^{\text{micro}}+\varepsilon }\text{.}$$

Macro-F1 is used as the primary model-selection and reporting metric because it weighs classes equally and is therefore sensitive to minority performance; micro-F1 is reported as a secondary aggregate. To isolate the contributions of algorithmic design choices, we predefine and execute the following ablations on the identical folds used above: (i) uniform averaging versus validation-weighted soft voting, (ii) with versus without class-weighted losses; with versus without training-fold oversampling, (iii) fixed threshold 0.5 versus calibrated per-class thresholds selected on validation, and (iv) leave-one-backbone-out to quantify marginal contribution of each backbone; optional diversity diagnostics.

#### Explainability method and validation

Explanations are intended for analysis and triage and are not a substitute for clinical judgment. Given an input sequence $x=\left\{t_{1},\ldots ,t_{L}\right\}$ and the model’s predicted class $c^{*}$, LIME approximates the local decision boundary around x with a sparse linear surrogate fitted on perturbed variants of x. We generate S perturbed texts by masking subsets of tokens using a masking token (for transformer models we use the model-appropriate mask). Each perturbed sample $x^{\prime }$ is weighted by a similarity kernel $K_{\kappa }\left(x,x^{\prime }\right)=\exp\left({-\text{dist}\left(x,x^{\prime }\right)}^{2}/\kappa^{2}\right)$, where dist counts masked tokens and κ controls locality. A weighted linear model g is fit to predict the target probability (or logit) of $c^{*}$ from a binary indicator vector over tokens; the resulting coefficients define token importances. We retain the top-K tokens as the explanation. Unless stated otherwise, we use S∈[5,000,10,000], kernel width κ tuned on a small validation subset, masking as single-token blanks, and K∈{5,10}.

LIME explanations are local and surrogate-based; they can vary with random sampling and perturbation design and may not perfectly reflect the internal nonlinearity of the fused model. Masking can also introduce distribution shift. We therefore accompany explanations with objective faithfulness and stability checks and provide a brief human sanity check. To evaluate whether highlighted tokens contribute to the model’s prediction, we compute deletion curves. Let $p^{\left(0\right)}\left(c^{*}\right)$ be the model probability (or logit) for class $c^{*}$ on the original input x. Sort tokens by LIME importance in descending order and iteratively delete the top-k tokens to obtain $x^{\left(k\right)}$ and $p^{\left(k\right)}\left(c^{*}\right)$. The deletion curve is presented in Equation 17. We summarize faithfulness by the area under the deletion curve using Equation 18, where larger values indicate greater faithfulness. Moreover, we report sufficiency and comprehensiveness using Equation 19, where $p^{\left(\text{keep}\;K\right)}$ denotes the model score when only the top-K tokens are kept and the remainder are masked, and $p^{\left(\text{drop}\;K\right)}$ denotes the score after dropping the top-K tokens. We assess sensitivity to LIME’s randomness by repeating the procedure with r different seeds and computing overlap@ K of important token sets and Kendall’s τ between importance rankings.

(Equation 17) $$\Delta p\left(k\right)=p^{\left(0\right)}\left(c^{*}\right)-p^{\left(k\right)}\left(c^{*}\right),k=1,\ldots ,K\text{.}$$

(Equation 18) $${\text{AUC}}_{\text{del}}=\frac{1}{K}\sum_{k=1}^{K}\Delta p\left(k\right)\text{,}$$

(Equation 19) $$\text{Suff}\left(K\right)=p^{\left(0\right)}\left(c^{*}\right)-p^{\left(\text{keep}\;K\right)}\left(c^{*}\right),\text{Comp}\left(K\right)=p^{\left(0\right)}\left(c^{*}\right)-p^{\left(\text{drop}\;K\right)}\left(c^{*}\right)\text{,}$$
