# Supplementary material for: Ensemble transformer with post-hoc explanations for depression emotion and severity detection
Source: iScience. 2026 Jan 5;29(2):114605. doi: 10.1016/j.isci.2025.114605 (PMC12860732; doi:10.1016/j.isci.2025.114605)
Supplement: Document S1. Data S1 [file mmc1.pdf]

iScience, Volume *29*

Supplemental information

Ensemble transformer with post-hoc explanations for depression emotion and severity detection

Sazzadul Islam, Rezaul Haque, Mahbub Alam Khan, Arafath Bin Mohiuddin, Md Ismail Hossain Siddiqui, Zishad Hossain Limon, Katura Gania Khushbu, S M Masfequier Rahman Swapno, Md. Redwan Ahmed, and Abhishek Appaji

S1. Data

Algorithm 1: DepTformer-XAI-SV: Validation-weighted soft voting with per-class threshold calibration.

Require: Dataset $D = \{(x_i, y_i)\}_{i=1}^{N}$; base classifiers $C = \{C_1, \dots, C_M\}$; metric $s(\cdot)$ (macro-F1 on validation); thresholds grid $\mathcal{T}$ for multi-label

Output: Final prediction $\hat{y}$ for input x

Procedure

Construct k stratified folds (iterative stratification for multi-label). For each fold $f = 1..k$:

1. Split into train $T^{(f)}$, validation $V^{(f)}$, and test $U^{(f)}$ (the fold's held-out part).
2. For each classifier C_j:
   - 2.1. Hyperparameter search on $T^{(f)}$; train with early stopping (validation macro-F1).
   - 2.2. Predict probabilities $P_j(\cdot)$ on $V^{(f)}$ and compute $s_j^{(f)} = s\left(C_j; V^{(f)}\right)$.
3. Compute normalized weights $w_j^{(f)} = s_j^{(f)} / \sum_{k=1}^{M} s_k^{(f)}$.
4. Multi-label only: choose per-class thresholds

$$\tau_c^{(f)} = \arg\max_{\tau \in \mathcal{T}} \mathrm{F1_c} \left(\mathbb{1} \left[\sum_j w_j^{(f)} P_j(y = c \mid x) \geq \tau \right] \text{ on } V^{(f)} \right).$$

5. For any input x (validation or test), form the fused probability

$$P_{\mathrm{ens}}(y = c \mid x) = \sum_{j=1}^{M} w_j^{(f)} \; P_j(y = c \mid x).$$

6. Decision: multi-class $\hat{y} = \arg\max_c P_{\mathrm{ens}} (y = c \mid x)$; multi-label $\hat{y_c} = \mathbb{1}\left[P_{\mathrm{ens}}(y = c \mid x) \geq \tau_c^{(f)} \right]$.
7. Evaluate on $U^{(f)}$ and record macro/micro metrics.
